# Microbial sensing in the intestine

**DOI:** 10.1093/procel/pwad028

**Published:** 2023-05-16

**Authors:** Tingting Wan, Yalong Wang, Kaixin He, Shu Zhu

**Affiliations:** Division of Life Sciences and Medicine, The CAS Key Laboratory of Innate Immunity and Chronic Disease, Institute of Immunology, School of Basic Medical Sciences, University of Science and Technology of China, Hefei 230027, China; Division of Life Sciences and Medicine, The CAS Key Laboratory of Innate Immunity and Chronic Disease, Institute of Immunology, School of Basic Medical Sciences, University of Science and Technology of China, Hefei 230027, China; Division of Life Sciences and Medicine, The CAS Key Laboratory of Innate Immunity and Chronic Disease, Institute of Immunology, School of Basic Medical Sciences, University of Science and Technology of China, Hefei 230027, China; Division of Life Sciences and Medicine, The CAS Key Laboratory of Innate Immunity and Chronic Disease, Institute of Immunology, School of Basic Medical Sciences, University of Science and Technology of China, Hefei 230027, China; Department of Digestive Disease, Division of Life Sciences and Medicine, The First Affiliated Hospital of USTC, University of Science and Technology of China, Hefei 230001, China; Institute of Health and Medicine, Hefei Comprehensive National Science Center, Hefei 230601, China

**Keywords:** mucosal immunology, pattern recognition receptors, protein-coupled receptors, intestinal epithelial cells, immune cells, gut microbiota, enteric viruses, inflammatory bowel disease

## Abstract

The gut microbiota plays a key role in host health and disease, particularly through their interactions with the immune system. Intestinal homeostasis is dependent on the symbiotic relationships between the host and the diverse gut microbiota, which is influenced by the highly co-evolved immune–microbiota interactions. The first step of the interaction between the host and the gut microbiota is the sensing of the gut microbes by the host immune system. In this review, we describe the cells of the host immune system and the proteins that sense the components and metabolites of the gut microbes. We further highlight the essential roles of pattern recognition receptors (PRRs), the G protein-coupled receptors (GPCRs), aryl hydrocarbon receptor (AHR) and the nuclear receptors expressed in the intestinal epithelial cells (IECs) and the intestine-resident immune cells. We also discuss the mechanisms by which the disruption of microbial sensing because of genetic or environmental factors causes human diseases such as the inflammatory bowel disease (IBD).

## Introduction

The human gut lumen contains trillions of microorganisms, including bacteria, viruses, fungi, and archaea. These microorganisms are collectively termed as the “microbiota” and harbor 100-fold more genes than the humans (Sommer and Bäckhed, 2013). The human microbiota is estimated to consist of nearly 4 × 10^13^ microbial cells, which is approximately equivalent to the total number of cells in the human body (Elson and Alexander, 2015; Sender et al., 2016a, 2016b). The Human Microbiome Project (HMP) was established in 2008 and aimed to (i) characterize the microbial communities found in different parts of the human body; (ii) identify the correlation between changes in the microbiome and the human health; and (iii) demonstrate opportunities to improve human health by manipulating the human microbiome (Group et al., 2009). The HMP initiative and other “omics” studies including metabolomics, transcriptomics, and proteomics, have significantly expanded our understanding of the diversity and complexity of the human microbiota (Palm et al., 2015). In the last 2 decades, lots of studies have shown that the microbiota-host crosstalk plays an important role in maintaining the intestinal homeostasis for both the host and the microbiota, and also plays a critical role in the systemic health, physiology, and development of the host. Current progress in the field has shown that the gut microbiota significantly impacts human health and disease. The host immune system recognizes antigens derived from the structural components of the microbiota and their metabolites and mount an optimal immune response to maintain the physiological functions of the host (Franzosa et al., 2015; Rooks and Garrett, 2016).

The three distinct inter-linked layers of the mammalian gastrointestinal tract, namely, (i) the luminal mucus layer; (ii) the 10-μm monolayer of intestinal epithelial cells; and (iii) the internal layer of the mucosal immune system called as the lamina propria, maintained a delicate balance between the immune system and the tissue homeostasis in the gut lumen (Mowat and Agace, 2014). The immune cells in the gut barrier such as the macrophages, dendritic cells (DCs), innate lymphoid cells (ILCs), T cells and B cells, and the non-immune intestinal epithelial cells (IECs) play a significant role in the gut–microbiota interactions. The pattern recognition receptors (PRRs) play a crucial role in the innate immune system and constitute a large collection of proteins that play a critical role in maintaining the immune-microbiota homeostasis by rapidly recognizing signaling molecules derived from the non-commensal microbes and the injured host intestinal tissue components (Medzhitov and Janeway, 2000; Burgueno and Abreu, 2020). For example, mutations in NOD2, a well-characterized PRR, is significantly associated with the inflammatory bowel disease (IBD) (Hugot et al., 2001; Ogura et al., 2001; Khor et al., 2011). The host cells also express receptors, which sense and respond to metabolites derived either from the anaerobic fermentation of undigested dietary components or from compounds that are *de novo* synthesized by the microbes or the host cells (Eshleman and Alenghat, 2021). The immune system-microbiota homeostasis is maintained by the activity of several signaling pathways. However, genetic mutations in the host genome, infections, antibiotics, and/or diet can disrupt the delicate host-microbiota balance causing dysbiosis, which contributes significantly to the pathogenesis of infections, autoimmune diseases, inflammatory diseases, obesity, cancer, and other diseases (Gopalakrishnan et al., 2018). In this review, we summarize the mechanisms by which the host intestinal cells sense the microbes and gut metabolites derived from the microbe to maintain gut homeostasis. We also describe the mechanisms by which disruption of the host-microbiota homeostasis in the gut adversely affects human health and disease.

## Sensing of microbes and metabolites by receptors in intestine

Skin and mucosa act as physical barriers and prevent the entry of pathogens such as bacteria, viruses, and fungi into the host. However, when these pathogens breach the first line of defense, the innate immune system immediately responds and produces antimicrobial products and recruits a wide variety of immune cells to eliminate them (Liu and Cao, 2016). Microbial ligands (e.g., nucleic acids and the bacterial cell wall components) are detected by the germline encoded PRRs to initiate downstream immune responses against the invading microbes. The microbial metabolites are recognized by the G protein-coupled receptors (GPCRs), aryl hydrocarbon receptors (AHRs), nuclear receptors, and other receptors, which modulate innate immune signaling pathways, impact epithelium integrity, and importantly, regulate hormonal signals release. Gut hormones have a wide range of targets in the whole body and play systemic physiological roles especially in the control of metabolism. Except for products of food digestion (e.g., glucose, amino acids, and fatty acids), microbial products [such as short-chain fatty acids (SCFAs), lipopolysaccharide (LPS) and secondary bile acids] also act as stimuli for local enteroendocrine cells (EECs) to generate hormonal signals that reflect dietary intake, microbial composition and epithelial integrity (Gribble and Reimann, 2019).

### Microbial pattern recognition by the intestinal PRRs

**Figure 1.**
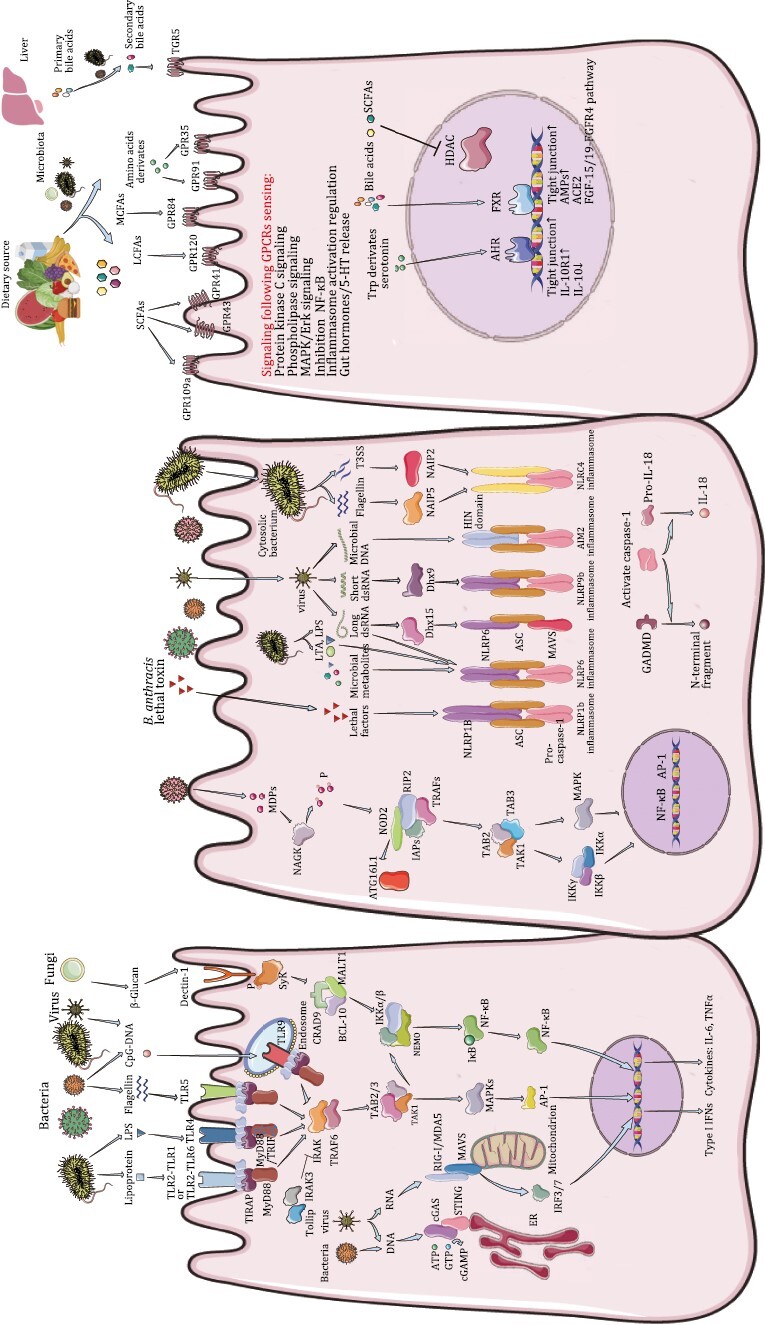
**Microbial signal–host sensor interactions in IECs and associated signaling events.** (Left) Microorganism-associated molecular patterns (MAMPs) from the bacteria, fungi and viruses are sensed by extensive types of pattern recognition receptors (PRRs), which are broadly expressed in/on intestinal epithelial cells (IECs). For toll-like receptors (TLRs), TLR4 senses bacterial lipopolysaccharide (LPS), TLR2-TLR1 or TLR2-TLR6 dimers sense bacterial lipoprotein, TLR5 senses bacterial flagellin and TLR9, which mainly localized on endosome membrane, is triggered by CpG DNA from both bacteria and viruses. Once binding ligands, TLR homologous or heterodimers are formed and MyD88-dependent and/or MyD88-independent signaling pathways are activated by different adaptor proteins (AP) such as MyD88, Toll/IL-1 receptor (TIR) domain-containing adaptor protein (TIRAP), TIR domain-containing adaptor molecule (TRAM) and/or TIR domain-containing adaptor inducing IFNβ (TRIF). However, TLR2 and TLR4 need TIRAP to recruit MyD88. TLR4 can initiate downstream signals using both MyD88 and TRIF as signaling adaptors, but it also needs TRAM to recruit TRIF and TIRAP to recruit MyD88. MyD88 attracts IL-1 receptor-associated kinases (IRAKs) to TLRs through the interaction of two molecular death domains and IRAK-1 is activated by phosphorylation, which then interacts with TNF-receptor-associated factor (TRAF), a ubiquitin ligase. Typically, TRAF6 forms complexes with TGF-beta activated kinase (TAK) and TAK1-associated binding protein 2 (TAB), and TAK activates downstream mitogen activated protein (MAP) kinase [Jun N-terminal kinases (JNK), p38 MAPK] and inhibitor of nuclear factor kappa-B kinase (IKK), leading to activation of nuclear factor kappa-B (NF-κB) and activator protein 1 (AP-1), and therefore regulating the expression of pro-inflammatory cytokines and other immune-related genes. TLR4 and TLR3 could induce TRIF-dependent IFN-I pathway, and activate similar downstream signals through TRAF3 or receptor-interacting serine/threonine-protein kinase 1 (RIPK1)/TRAF6. Both Tollip and IRAK-3 interact with IRAK-1 and negatively regulate TLR-mediated signal transduction pathways. Viruses are sensed by retinoic acid-inducible gene I (RIG-I), which detect 5ʹ-ppp RNA and short-chain dsRNA, and melanoma differentiation-associated gene 5 (MDA5), which detect long-chain dsRNA respectively, and lead to type I IFN production in a mitochondrial antiviral signaling protein (MAVS)- and stimulator of interferon response cGAMP interactor (STING)-dependent manner. DNA viruses activate the cyclic GMP-AMP synthase (cGAS)-STING pathway. In the presence of DNA, cGAS enzymatically converts ATP and GTP into the cytosolic dinucleotide, cGAMP, which then binds to STING and activates IFN signaling. Dectin-1, officially named as C-type lectin domain-containing 7A (CLEC7A), a member of the C-type lectin family, senses β-glucans from fungi and promotes spleen tyrosine kinase (SyK) phosphorylation and subsequent signaling activation. (Mid) NLRs are a subset of cytoplasmic PRRs. On sensing their respective peptidoglycan ligands [d-glutamyl-meso-diaminopimelic acid (DAP) or phosphorylated muramyl dipeptide (MDP)], nucleotide-binding oligomerization domain-containing 1 (NOD1) and NOD2 undergo auto-oligomerization and induce NF-κB signaling by activating receptor-interacting serine/threonine kinase 2 (RIP2). Or NODs can interact with autophagy related 16 like 1 (ATG16L1) to induce autophagy. ATG16L1 is also a negative regulator of NOD signaling. NOD-like receptors (NLRs) such as NLRP1b, NLRP6 and NLRP9b oligomerize upon activation to form inflammasome complexes, which serve as platforms for the recruitment, cleavage, and activation of the inflammatory Caspase-1 (CASP-1). Subsequently, activated CASP-1 can process pro-IL-1β, pro-IL-18 or gasdermin D (GSDMD) into active forms, which in turn stimulate cytokine secretion or pyroptosis. Furthermore, NLRP6 can sense and bind the long viral dsRNA directly or through the RNA helicase DEAH-box helicase 15 (DHX15), which forms liquid–liquid phase separation (LLPS) to integrate inflammasome and and ISG. Microbial metabolites such as SCFAs could regulate NLRP6 inflammasome function. NLRP9b senses and binds the short viral dsRNA through DHX9 and activates the inflammasome. Flagellin and PrgJ, a conserved type III secretion system (TTSS) rod component from bacteria, bind to their corresponding adaptors, NAIP5 and NAIP2, respectively. Subsequently, they interact with NLRC4 to initiate formation of the NLRC4 inflammasome. (Right) Microbiota-processed metabolites could be sensed mainly by G Protein-Coupled Receptors (GPCRs) on cell membrane or by aryl hydrocarbon receptor (AHR) and nuclear receptors in cell nucleus and induce downstream signaling pathways. GPR41/43/109a are part of typical receptors for short-chain fatty acids (SCFAs, <6C), GPR120 could sense long -chain fatty acids (LCFAs, >12C) and GPR84 is stimulated by medium-chain fatty acids (MCFAs, 6C~12C). SCFAs could directly inhibit the activity of Histone deacetylase (HDAC). Amino acids derivates such as tryptophan and tyrosine catabolism products could activate GPR35, and succinate binds GPR91. Tryptophan derivatives, for example, indole-3-aldehyde could also stimulate nuclear AHR. Primary bile acids are produced in liver and some of them would be metabolized into secondary bile acids by microbiota when come to distal intestine. Nuclear receptor farnesoid X-activated receptor (FXR) can bind bile acids and regulate bile acid metabolism. G protein-coupled bile acid receptor 1 (GP-BAR1, also named TGR5) is a membrane GPCR and mainly binds to secondary bile acids.

The microbial pattern recognition model was first proposed by Charles Janeway Jr. It has since been established through the discovery and characterization of numerous PRRs (Janeway and Medzhitov, 2002). PRRs are classified into five families based on their protein domain homology—(i) Toll-like receptors (TLRs), (ii) NOD-like receptors (NLRs), (iii) retinoic acid-inducible gene I (RIG-I)-like receptors (RLRs), (iv) C-type lectin receptors (CLRs), and (v) cytosolic DNA sensors including absence in melanoma 2 (AIM2)-like receptors (ALRs) and cyclic GMP-AMP synthase (cGAS). PRRs recognize evolutionarily conserved pathogen-associated molecular patterns (PAMPs) such as LPS, flagellin, bacterial DNA, and bacterial RNA. PRRs also sense danger-associated molecular patterns (DAMPs) according to the “danger theory” proposed by Matzinger (2002). DAMPs include molecules such as ATP, high mobility group box 1 protein (HMGB1), and uric acid, which are released from the injured cells. They subsequently bind to the PRRs and activate specific downstream signaling pathways. The activation of PRR signaling by PAMPs or DAMPs plays a significant role in pathogen clearance and tissue homeostasis. In this section, we review the intestinal PRRs, including (i) specific cell membrane-expressed TLRs, which respond to the extracellular microbes; (ii) TLRs in the endosomes or lysosomes, which regulate inflammatory cytokine production; and (iii) cytoplasm-expressed NLRs or RLRs, which respond to microbes or components, enter the nucleus to induce transcription of genes encoding cytokines such as interleukin (IL)-1β and IL-18, and trigger inflammatory cell death termed as “pyroptosis” (Fig. 1).

### TLRs

TLRs are type I transmembrane receptors that are characterized by an extracellular leucine-rich repeat (LRR) domain and an intracellular Toll/IL-1 receptor (TIR) domain (Rock et al., 1998). In the PRRs, LRR domain is responsible for ligand recognition and the TIR domain is required for signal transduction. Ten functional TLRs (TLR1–10) have been reported in humans and 12 TLRs (TLR1–9 and TLR11–13) have been reported in mice (Chuang and Ulevitch, 2001; Temperley et al., 2008; Mathur et al., 2016; Li and Wu, 2021). TLRs play a key role in shaping the intestinal microbiota and maintaining gut homeostasis. Myeloid differentiation factor 88 (MyD88) is an adaptor protein (AP) that is used by most TLRs and MyD88-deficent mice demonstrate defective T cell function and aggravated colitis (Takeuchi and Akira, 2010; Pandey et al., 2014; Wang et al., 2015b). TLR3 mediates transcriptional induction of type I interferons (IFNs), pro-inflammatory cytokines, and chemokines through the TIR domain-containing adaptor inducing IFNβ (TRIF) dependent pathway; TLR4 activates both MyD88- and TRIF-dependent pathways (Takeuchi and Akira, 2010; Pandey et al., 2014). MyD88-dependent microbial sensing is required to maintain the intestinal epithelial homeostasis and the production of antimicrobial peptides in the intestine (Rakoff-Nahoum et al., 2004; Vaishnava et al., 2008; Menendez et al., 2013; Bhinder et al., 2014).

TLRs are expressed in different cell types of the gut, including the IECs, immune cells, stromal cells, and the neuronal cells (Burgueño et al., 2016; Price et al., 2018; Burgueno and Abreu, 2020). The expression pattern of 5 TLRs (TLR2, TLR4, TLR5, TLR7, and TLR9) in the gut epithelium was established by experiments with the TLR-specific fluorescent reporter mice (Price et al., 2018). The expression levels of TLRs 6–9 and 11–13 were significantly low in both the small intestinal (SI) and the colonic IECs; the expression levels of TLRs 1, 2, 4, and 5 were moderate to low in the SI but significantly high in the colonic IECs; TLR3 expression levels were comparable in the SI and the colonic enterocytes (Price et al., 2018). IECs of the specific pathogen free (SPF) mice showed higher expression levels of most TLRs compared to those from the germ-free mice (Hörmann et al., 2014; Huhta et al., 2016; Fang et al., 2022). These data suggested that the expression levels of TLRs in the IECs were associated with the increased abundances of the commensal microbes.

TLR2 shows constitutive expression in the gastrointestinal epithelium and the enteric neurons, and mononuclear cells in the lamina propria even could release soluble forms of the TLR2 ectodomain, especially in patients with enteric inflammation (Aliprantis et al., 1999; Candia et al., 2012; Burgueño et al., 2016). The heterodimers of TLR1 or TLR6 with TLR2 (TLR1/TLR2 or TLR6/TLR2) on the plasma membrane recognized small-molecule agonists such as lipoteichoic acid (LTA) and lipopeptide (Cheng et al., 2015; Li and Wu, 2021). Pam3CSK4 is a synthetic tri-acylated lipopeptide that can activate TLR2. Dextran sulfate sodium (DSS)-induced colitis model mice pre-treated with Pam3CSK4 showed lower body weights and rectal bleeding (Horuluoglu et al., 2020). Pam3CSK4 stimulation of TLR2 effectively preserved the intestinal epithelial tight junction (TJ)-associated barrier assembly against stress-induced damage by inducing Phosphatidylinositol-3-kinase (PI3K)/Akt-mediated cell survival via MyD88 (Cario et al., 2007). TLR2-deficient mice showed severe chemotherapy-induced SI mucositis that was characterized by the accumulation of damaged DNA and infiltration of the CD11b^+^ myeloid cells in the proximal jejunum (Frank et al., 2015). Moreover, wild-type mice treated with antibiotics to deplete gut microbiota showed increased susceptibility to chemotoxicity, but the chemo-toxic effects were alleviated by pre-treatment with a TLR2 agonist (Frank et al., 2015). The abrogation of epithelial TLR2 signaling impaired mucosal repair because of reduced numbers of goblet cells (Podolsky et al., 2009; Walker et al., 2022). Diacyl phosphatidylethanolamine with two branched chains (a15:0-i15:0 PE) derived from *Akkermansia muciniphila* exhibited immunomodulatory selectivity and weak immunoregulatory effects through the non-canonical TLR2–TLR1 signaling pathway (Bae et al., 2022). Moreover, genetic ablation of TLR2 disrupted the structure and function of the enteric nervous system (ENS) in mice (Brun et al., 2013; Burgueño et al., 2016; Schill et al., 2022). The stimulation of colonic TLR2 signaling by gram-positive bacteria promoted colonic neurogenesis to maintain the nitrergic neurons in the longitudinal muscle myenteric plexus (Yarandi et al., 2020). These data demonstrated that sensing of microbial lipopeptides by TLR2 was critical for maintaining the integrity of the intestinal epithelium, ENS function, and immune tolerance to the bacteria.

TLR4 is a well-characterized PRR that is expressed on the cell membrane and the endosomes of the immune cells, enteric neurons, and the enterocytes (Neal et al., 2006). Activation of TLR4 on the cell surface by the extracellular LPS derived from the outer membrane of the Gram-negative bacteria mediates the downstream immune responses (Lu et al., 2022). For LPS cytosolic recognition, outer membrane vesicles produced by the Gram-negative bacteria could deliver LPS into the cytosol and trigger caspase 11-dependent effector responses (Shi et al., 2014; Vanaja et al., 2016). TLR4 expression is increased in the IECs during intestinal inflammation such as IBD; moreover, increased TLR4 signaling disables host protection against intestinal inflammation (Hausmann et al., 2002; Neal et al., 2006; Yang et al., 2017). A high-fat diet (HFD) causes gut microbiota dysfunction and exacerbates intestinal inflammation through the TLR4 signaling pathway (Kim et al., 2012). Conversely, in the murine acute colitis model, TLR4 deficiency caused severe mucosal damage, which was characterized by impaired epithelial proliferation and significant bacterial invasion (Fukata et al., 2005). Furthermore, TLR4 protected against intestinal inflammation by modulating the inhibitory immune responses associated with *A. muciniphila* (Liu et al., 2022b). TLR4 also regulated the gut microbiota by altering gastrointestinal motility to drive pathogen clearance, thereby regulating maintenance of the commensal microbiota, goblet cell differentiation, and production of the antimicrobial peptides (Vora et al., 2004; Sodhi et al., 2012). TLR4 demonstrates two-sided function during bowel inflammation which looks like to result from distinct TLR4 responses induced by different microbial species. TLR4 is expressed on the intestinal stem cells (ISCs) and induced stem cell apoptosis through innate immune signaling and therefore inhibits their proliferation (Neal et al., 2012; Afrazi et al., 2014; Hackam and Sodhi, 2022). However, hyperactivation of TLR4 triggered a neoplastic developmental program in the mouse models (Santaolalla et al., 2013). Furthermore, epithelial TLR4 deficiency increased the abundance of goblet cells and suppressed Notch signaling in the SI IECs (Sodhi et al., 2012; Hackam and Sodhi, 2022). LPS-triggered TLR4 signaling regulates the intestinal inflammatory responses and modulated the crypt stem cell functions, but the regulation differs under different circumstances. Similar to TLR2, TLR4 was also expressed in the gut neuronal cells and played a key role in the gut neuron cell signaling and development (Anitha et al., 2012; Kovler et al., 2021; Schill et al., 2022; Bódi et al., 2023).

TLR5 is localized on the cell surface and is highly expressed in the crypts of the small intestine and IECs of the colon; it is activated by bacterial flagellins (Gewirtz et al., 2001; Price et al., 2018). A screening study of flagellins in the human gut metagenomes that recognize and activate TLR5 resulted in the discovery of a class of silent flagellins, which were produced by the gut bacteria called *Lachnospiraceae*; silent flagellins induced weak TLR5 activation and innate immune tolerance (Clasen et al., 2023). X-linked inhibitor of apoptosis protein (XIAP)-mediated resilience of TLR5 signaling in the TLR5-expressing Paneth cells and the intestinal DCs enabled them to maintain tissue integrity and microbiota homeostasis during ileitis (Wahida et al., 2021). Null or epithelial-specific deletion of TLR5 impaired pathogen clearing and increased the susceptibility to microbial-induced colitis and metabolic syndrome (Vijay-Kumar et al., 2010; Letran et al., 2011; Chassaing et al., 2014). This suggested that dysfunction of the innate immune system promoted development of the metabolic syndrome. Mice with TLR5 deficiency showed increased body mass at 20 weeks of age. Magnetic resonance imaging (MRI) data demonstrated increased visceral and epididymal fat mass in the TLR5-deficient mice and correlated with increased serum triglycerides and high blood pressure. HFD-fed TLR5-deficient mice exhibited inflammatory infiltrates in the pancreatic islets and hepatic steatosis, but this phenotype was reversed by depleting the gut microbiota using broad-spectrum antibiotics. Moreover, germ-free mice transplanted with microbiota from the TLR5-deficient mice showed various symptoms of metabolic syndrome such as obesity, insulin resistance, hyperglycemia, and elevated levels of pro-inflammatory cytokines, which were characteristic features of the TLR5-deficient mice. These results suggested that the altered gut microbiota in the TLR5-deficient mice contributed to the development of the metabolic syndrome (Vijay-Kumar et al., 2010). In the small intestine, TLR5 expression was restricted to the Paneth cells and gradually decreased during the neonatal period (Price et al., 2018). TLR5 recognition of the flagellin from *Roseburia intestinalis* upregulated the TJ protein Occludin and restored the gut microbiota through elevated expression of IL-22 and REG3γ (Fulde et al., 2018; Seo et al., 2020). Moreover, TLR5 activation promoted early defense mechanisms against pathogen invasion of the host tissues by inducing the production of IL-17 and IL-22 on the mucosal surface (Frosali et al., 2015; Song et al., 2016). TLR5/NCLR-4-mediated synthesis of IL-22 and IL-18 during bacterial flagellin treatment was critical for the prevention and cure of rotavirus (RV) infection in mice (Zhang et al., 2014a). However, TLR5-deficient mice were resistant to *Salmonella* infection. The small intestine and colon tissues in the TLR5-deficient mice showed upregulated levels of the host defensive genes, antimicrobial peptides, and the serum and fecal Immunoglobulin A (IgA) (Uematsu et al., 2006; Vijay-Kumar et al., 2008a). TLR5 was also unexpectedly found to be co-expressed with the neurofilament-200 in the large-diameter A-fiber neurons of the dorsal root ganglion (DRG). Activation of TLR5 with its flagellin ligand induced TLR5-dependent blockade of the sodium currents, predominantly in the A-fiber neurons of the DRG and silenced the Aβ-fibers (Xu et al., 2015). Together, these studies demonstrated that TLR5 played a significant role in the intestinal pathogen defense and inflammation. TLR5 deficiency correlated with metabolic dysfunction. Further applications of the TLR5 ligand flagellins require in-depth investigations.

Other TLRs have also been reported to play significant roles in the host–microbiota interactions. Mice pre-treated with the TLR3 agonist poly(I:C) and the TLR7 agonist, imiquimod, were protected from DSS-induced colitis, whereas mice deficient in both TLR3 and TLR7 were more susceptible to DSS-induced colitis. A genome-wide association study (GWAS) showed that rs3775291 in TLR3 was significantly associated with susceptibility to both ulcerative colitis (UC) and Crohn’s disease (CD). This was consistent with the results from animal studies. Human subjects with combined TLR3 and TLR7 genetic variations showed severe UC, with a higher cumulative rate of hospitalization for patients with two combined genetic variants (rs3775291 in TLR3 and rs3853839 in TLR7) than those with or without a single variant (Yang et al., 2016). In the bat intestinal organoids, rapid and robust induction of the TLR3 response induced the bat cells to prevent virus propagation in the early phase of infection (Liu et al., 2022a). Several studies have reported that majority of the TLRs are localized to the basolateral membrane, whereas TLR3 and TLR9 are mostly located at the apical surface (Yu and Gao, 2015; Price et al., 2018). Viral infections from the basolateral side of the IECs elicit a stronger intrinsic immune response in comparison to the luminal apical infections; moreover, clathrin-sorting AP-1B mediated polarized sorting of the TLR3 towards the basolateral side of the IECs (Stanifer et al., 2020). In the colonocytes, basolateral TLR9 signaling induced IκBα degradation and activation of the nuclear factor kappa-B (NF-κB) pathway, whereas apical TLR9 stimulation invoked a unique response in which ubiquitinated IκB accumulated in the cytoplasm and prevented NF-κB activation (Lee et al., 2006). TLR9 also showed a protective function in the DSS colitis model; TLR9-deficient mice showed decreased expression levels of the intestinal repair genes such as hairy enhancer of split 1 and the vascular endothelial growth factor (Rose et al., 2012). TLR9 also induced intestinal inflammation against pathogens by sensing the bacterial and viral CpG DNA (Chen et al., 2020b; Zhong et al., 2022).

### NLRs

NLRs are a group of cytosolic receptors that mediate caspase-1 activation, secretion of the pro-inflammatory cytokines such as IL-1β and IL-18, and inflammatory cell death in response to various cytoplasmic stimuli (de Zoete et al., 2014). NLRs consist of (i) a ligand-binding LRR domain similar to the TLRs, (ii) a central nucleotide-binding domain (NACHT) that is involved in oligomerization of the NLRs, (iii) a N-terminal interaction domain for signal transduction to activate downstream target proteins such as the caspases, and (iv) a caspase recruitment domain (CARD) or a pyrin domain (PYD) (Fritz et al., 2006; Kim et al., 2016; Eshleman and Alenghat, 2021). Recent studies have reported that NLRs play a critical role in maintaining intestinal homeostasis by responding to the intestinal microbes or their derivatives (de Zoete and Flavell, 2013; Guo et al., 2020).

Both nucleotide-binding oligomerization domain-containing 1 (NOD1) and NOD2 sense cytosolic bacterial peptidoglycan fragments. NOD1 senses d-glutamyl-meso-diaminopimelic acid (DAP)-containing peptidoglycan fragments, which are mainly found in the Gram-negative bacteria, whereas NOD2 responds to muramyl dipeptide (MDP), which is found in all the bacterial strains (Chamaillard et al., 2003; Girardin et al., 2003a, 2003b). The results of a forward genetic screening experiment to identify factors required for MDP detection showed that *N*-acetylglucosamine kinase (NAGK) was essential for the immunostimulatory activity of MDP and NAGK-phosphorylated MDP was an agonist for NOD2 (Stafford et al., 2022). NOD2 used the AP mitochondrial antiviral signaling protein (MAVS) after recognizing the viral single-stranded RNA (ssRNA) to activate IRF3 and induced IFN-β synthesis and secretion (Sabbah et al., 2009). Both NOD1 and NOD2 are expressed in the gut-resident DCs, macrophages, and the IECs; NOD1 is constitutively expressed in the IECs, whereas NOD2 expression is restricted to the Paneth cells in the small intestine (Lala et al., 2003; Eshleman and Alenghat, 2021). In mice, the recognition of Gram-negative bacteria by NOD1 in the epithelial cells was necessary and sufficient to induce genesis of the isolated lymphoid follicles (ILFs) (Bouskra et al., 2008). NOD1 ligands derived from the intestinal bacteria also acted as circulating signal molecules and directly modulated insulin trafficking in the pancreatic beta cells via sensing by NOD1 and its downstream adaptor, receptor-interacting-serine/threonine-protein kinase 2 (RIP2) (Zhang et al., 2019). NOD2 activation in the Paneth cells by the microbial peptidoglycans induced cytokine release, antimicrobial peptide production, autophagy, and epithelial cell regeneration (Nigro et al., 2014). NOD2-deficient mice inoculated with *Helicobacter hepaticus* induced ileal granulomatous inflammation, but this phenotype was reversed by the transgenic expression of α-defensins in the Paneth cells (Biswas et al., 2010). However, multiple studies reported contradictory results suggesting that NOD2 was not a direct regulator of the antimicrobial function of the Paneth cells. These studies demonstrated that the previously reported NOD2-dependent changes in the gut microbial composition could be overcome by environmental factors such as co-housing with the wild-type littermates (Petnicki-Ocwieja et al., 2009; Shanahan et al., 2014; Wilson et al., 2015b). NOD2 deficiency caused a pro-inflammatory environment because of dysbiosis and increased the risk of colitis and colitis-associated carcinogenesis, but this risk was reduced by transplanting normal microbiota (Couturier-Maillard et al., 2013). Moreover, the expression of NOD2 was dependent on the gut microbiota. In the germ-free mice, NOD2 expression was significantly reduced in the terminal ileum, but was re-induced by colonization with the commensal microbiota (Petnicki-Ocwieja et al., 2009). Furthermore, GWAS and functional screening studies showed that NOD2 polymorphisms were significantly associated with IBD susceptibility in humans (Hugot et al., 2001; Ogura et al., 2001; Tan et al., 2015; Ntunzwenimana et al., 2021). As a follow-up of these reports, several studies have investigated mechanisms underlying the induction of intestinal inflammation and dysbiosis by NOD2 in patients with CD. NOD2 promoted IgA transport through the human and mouse microfold cells (M cells) by downregulating the expression of two retrograde transport receptors, Dectin-1 and Siglec-5; moreover, secretory IgA (sIgA) transport in the *NOD2*-mutated CD patients significantly increased compared to the CD patients without *NOD2* mutations or healthy subjects (Rochereau et al., 2021). NOD2 also induced microbiota dependent endoplasmic reticulum (ER)-stress-mediated regulation of mucus secretion in the goblet cells and the ER stress-mediated antimicrobial pathway in the macrophages (Ranjan et al., 2021; Naama et al., 2023). Conversely, NOD2 induced the early IL-33-dependent expansion of group 2 ILCs during CD pathogenesis (De Salvo et al., 2021). In patients with NOD2-driven CD, gp130 blockade rescued the activated inflammatory program in the NOD2-deficient cells and complemented anti-tumor necrosis factor (TNF) therapy (Nayar et al., 2021). This demonstrated that NOD2 was significantly associated with IBD by sensing bacterial MDP and modulated the antimicrobial cell function. NOD2 also played a key role in the metabolic syndrome. NOD2 in the IECs was necessary for the production of insulin-like growth factor-1 (IGF-1) mediated by a strain of *Lactiplantibacillus plantarum* (strain Lp^WJL^) and also promoted postnatal growth in the malnourished animals (Schwarzer et al., 2023). This suggested that the bacteria cell wall components or purified NOD2 ligands (MDP and mifamurtide) may alleviate growth stunting.

NLR family, CARD domain-containing 4 (NLRC4) is an N-terminal CARD domain-containing NLR that discriminates commensal bacteria from the pathogens (Franchi et al., 2012; Nordlander et al., 2014). NLRC4 is highly expressed in the intestinal mononuclear phagocytes and the epithelial crypts. NLR family apoptosis inhibitory protein (NAIP)-NLRC4 inflammasomes coordinated the expulsion of IECs through the release of eicosanoids and IL-18 via activation of caspases-1 and -8 (Rauch et al., 2017). Pathogenic Gram-negative bacteria such as *Salmonella* and *Pseudomonas* promoted the release of NLRC4-dependent IL-1β by stimulating the intestinal mononuclear phagocytes. However, the release of IL-1β was not observed when the intestinal mononuclear phagocytes were stimulated by the commensal bacteria such as *Bacteroides fragilis*, *Enterococcus faecalis* and *Lactobacillus* (Franchi et al., 2012). NLRC4 responded to the invading pathogens by sensing flagellin and the bacterial Type III secretion systems (T3SS) needle protein PrgJ/CprI (Franchi et al., 2006; Miao et al., 2006, 2010; Molofsky et al., 2006; Kofoed and Vance, 2011; Zhao et al., 2011). In the cytosol, flagellin and PrgJ/CprI interacted with NLRC4 to initiate oligomerization by binding to the APs NAIP2 and NAIP5, respectively, and activated the inflammasomes (Conforti-Andreoni et al., 2011; Kofoed and Vance, 2011; Zhao et al., 2011). Flagellin also promoted immediate elimination of the RV-infected cells via induction of the NLRC4-dependent IL-18 (Zhang et al., 2014a, 2020). NLRC4 protected against DSS-induced experimental colitis and azoxymethane/dextran sodium sulfate (AOM/DSS)-induced tumorigenesis by regulating epithelial cell proliferation and apoptosis during injury (Hu et al., 2010; Nordlander et al., 2014). In mice challenged with *Burkholderia thailandensis* and *Salmonella typhimurium*, NLRC4 was activated by a specific constituent of the murine intestinal microbiota, *Escherichia coli* O21:H+; moreover, challenge induced translocation of *E*. *coli* O21:H+ to the white adipose tissue (WAT) sustained systemic insulin and IGF-1 signaling, thereby promoting disease tolerance and protection against wasting (Schieber et al., 2015). In summary, the sensing of flagellin and T3SS rod proteins from the pathogenic bacteria or the commensal bacteria by NLRC4 initiates immunogenic or immunotolerant responses.

NLR family, pyrin domain-containing 3 (NLRP3) is one of the best characterized NLRs to date. NLRP3 senses numerous ligands, including ATP, pore-forming toxins such as nigericin and viroporins, uric acid crystals, amyloid-β (Aβ) depositions, asbestos, silica particles, and nucleic acids (Yang et al., 2019; Coll et al., 2022; Harapas et al., 2022). Activated NLRP3 oligomerizes, binds and activates the ASC complex, and reactivates the effector complex composed of CARD and caspase-1. This NLRP3–ASC–pro-Caspase-1 complex is denoted as the NLRP3 inflammasome, which activates critical pro-inflammatory factors (Yang et al., 2019; Li and Wu, 2021). In select patients with active IBD, unfermented dietary β-fructan fibers such as oligofructose (FOS) ~8 sugars induced the pro-inflammatory cytokines by activating NLRP3- and TLR2-mediated signaling pathways (Singh et al., 2019b; Armstrong et al., 2023). *In vivo* mouse studies have demonstrated that NLRP3 is a key player in maintaining the mucosal barrier. For example, NLRP3-deficient mice were highly susceptible to DSS-induced colitis because of impaired epithelial integrity in the colon (Zaki et al., 2010; Hirota et al., 2011). Several studies demonstrated that hyperactivation of the NLRP3 inflammasome adversely impacted gut epithelial cell proliferation and intestine–blood barrier integrity (Zaki et al., 2010; Huang et al., 2019; Cui et al., 2020). However, lower levels of pro-inflammatory cytokines in the colon tissues of the NLRP3-deficient mice suggested that decreased NLRP3 inflammasome activation is required for amelioration of acute and chronic intestinal inflammation (Bauer et al., 2010; Wang et al., 2018b; Mehto et al., 2019; Sanchez-Lopez et al., 2019). These contrasting results may be due to differences in the experimental approaches between different studies. However, NLRP3 is closely associated with IBD. Several GWAS studies have identified single nucleotide polymorphisms (SNPs) in the *NLRP3* (rs35829419, rs10733113, rs4925648, and rs10925019), which are associated with CD (Schoultz et al., 2009; Villani et al., 2009; Cummings et al., 2010; Roberts et al., 2010). Furthermore, mice carrying the *Nlrp3*^*R258W*^ mutation maintained gut homeostasis in the cryopyrin-associated periodic syndrome (CAPS) model (Yao et al., 2017). CAPS disease-associated mutations are mostly located in the NACHT domain, but few mutations are located in the LRR domain of NLRP3 (Touitou et al., 2004; Booshehri and Hoffman, 2019; Zhou et al., 2021).

NLRP6 also plays a significant role in the intestine and is predominantly expressed in the enterocytes and the goblet cells (Chen et al., 2011; Elinav et al., 2011; Wlodarska et al., 2014). NLRP6 sensed viral RNA, microbiota-associated metabolites, bacteria cell wall component LTA, and LPS (Levy et al., 2015; Wang et al., 2015a; Hara et al., 2018; Leng et al., 2020; Shen et al., 2021). NLRP6 can also form an inflammasome (Li et al., 2022a). Microbiota-related metabolites such as taurine, histamine, and spermine stimulated the formation of an ASC-dependent NLRP6 inflammasome, which induced synthesis and secretion of the downstream pro-inflammatory cytokines (Levy et al., 2015). Stimulation of the NLRP6 inflammasome by the SCFAs protected against intestinal epithelial barrier impairment and abrogated high-fructose diet-induced hippocampal neuroinflammation and neuronal loss (Li et al., 2019a). The activation of NLRP6 resulted in the formation of specks in the cells (Ghimire et al., 2018; Hara et al., 2018). Interaction with the NLRP6 agonists and double-stranded RNA (dsRNA) caused NLRP6 to undergo liquid–liquid phase separation (LLPS), an essential step for recruiting other components of the inflammasome (Shen et al., 2021). The intrinsically disordered poly-lysine sequence (K350-354) of NLRP6 was required for the formation of the NLRP6 puncta upon interaction with the dsRNA (Shen et al., 2021). And further investigation is still needed to solve problems like how is dsRNA/NLRP6 LLPS physiologically or pathologically regulated in cells (Li et al., 2022a). NLRP6 plays a crucial role in regulating inflammation and host defense against microorganisms in the intestine. NLRP6 deficiency in the mouse colonic epithelial cells decreased the production of IL-18 and altered the composition of the fecal microbiota, with increased representation of *Prevotellaceae* and *TM7*. NLRP6-deficient mice showed spontaneous intestinal hyperplasia, inflammatory cell recruitment, and exacerbated DSS-induced colitis. Further antibiotic treatment and electron microscopy studies revealed the role of *Prevotellaceae* in the microbiota-associated colitis disease (Elinav et al., 2011). De-ubiquitination of the NLRP6 inflammasome by CYLD lysine 63 deubiquitinase (CYLD) decreased IL-18 levels in the colonic mucosa and regulated intestinal inflammation (Mukherjee et al., 2020). However, contradictory reports have shown that both wild-type and Nlrp6^−/−^ littermate mice display comparable sensitivity to DSS-induced colitis, (Lemire et al., 2017; Mamantopoulos et al., 2017). This suggested that NLRP6 did not influence the composition of the commensal gut microbiota. The host NLRP6 also aggravated the gastrointestinal graft-versus-host disease (GVHD) independent of the gut microbial composition (Toubai et al., 2019). Furthermore, NLRP6-deficient goblet cells showed defective mucus secretion into the lumen of the large intestine because of aberrant autophagy and inflammasome activation (Wlodarska et al., 2014; Birchenough et al., 2016). However, another study showed that the basal inner mucus layer formation and function was independent of the inflammasome activity and was determined by the gut microbiota (Volk et al., 2019). NLRP6 plays a vital role in the defense mechanisms against viral infection in the intestine. Systemic challenge of NLRP6-deficient and control mice with the encephalomyocarditis virus (ECMV; transmitted through the oral-fecal route) showed similar mortality rates but the viral loads in the gastrointestinal tract of the NLRP6-deficient mice were higher compared to the control mice. Moreover, NLRP6-deficient mice showed higher mortality and viremia compared to the control mice when challenged by ECMV or murine norovirus (MNV) through oral administration. Mechanistically, NLRP6 binds to the viral RNA through DEAH-box helicase 15 [DHX15, an RNA helicase, and subsequently interacted with MAVS to induce the synthesis of type I/III IFNs and IFN-stimulated genes (ISGs) (Wang et al., 2015a)]. NLRP6 also modulated the death program in the intrinsic enteric-associated neurons, including the microbe-responsive subset of viscerofugal CART^+^ (cocaine- and amphetamine-regulated transcript) neurons, which are enriched in the ileum and the colon (Matheis et al., 2020; Muller et al., 2020). Microbiota depletion caused impaired glucose regulation because of NLRP6- and caspase 11-dependent loss of the CART^+^ neurons (Muller et al., 2020). In summary, NLRP6 is activated by the bacteria and the viruses. It functions in the enterocytes, goblet cells, and the ENS by regulating pathogen defense, intestine inflammation, and neuronal death.

Like NLRP6, NLRP9b is also specifically expressed in the IECs and is associated with protection against rotavirus infection. Rotavirus is another type of enteric virus with dsRNA that directly infects the IECs and causes severe diarrheal illness. In contrast to NLRP6, NLRP9b sensed the short double-stranded RNA generated during replication of the rotavirus dsRNA outside the viroplasm through DHX9 and activated the inflammasome, which in turn induced IL-18 secretion and pyroptosis to restrict rotavirus replication (Zhu et al., 2017b). NLRP9b and NLRP6 recognize different viral RNA patterns. Therefore, the intestinal regions in which NLRP9b is expressed are different from those expressing NLRP6. Therefore, these two IEC-specific NLRs cooperatively protected the host against enteric viral infections (Li and Zhu, 2020).

NLRP10 is a member of the NLR family that lacks pathogen sensing ability because it does not contain a putative LRR domain (Wang et al., 2004). NLRP10 function was elusive for more than a decade. NLRP10 was considered as an anti-inflammatory NLR that inhibited ASC-caspase 1 activation (Imamura et al., 2010). A recent study showed that NLRP10 monitored mitochondrial integrity in an mtDNA-independent manner and assembled the inflammasome in the differentiated human keratinocytes (Próchnicki et al., 2023). A recent study showed that NLRP10 played a protective role in intestinal inflammation (Zheng et al., 2023). In the intestine, NLRP10 was expressed in the distal colonic IECs. NLRP10 mRNA levels were higher in the GF mice than in the SPF mice. This suggested that the microbiome regulated NLRP10 expression. Furthermore, NLRP10 forms an ASC-dependent inflammasome. Whole-body or conditional IEC depletion of NLRP10 reduced caspase-1 activation in the IECs and increased the susceptibility to DSS-induced colitis. In conclusion, NLRP10 is a potential target for treating autoinflammatory diseases. However, the regulation of NLRP10 expression by the microbiota requires further investigation.

NLRP12 is expressed in the immune cells. However, the ligands of NLRP12 are unknown. NLRP6 and NLRP12 demonstrate dual functions. They can function both as an inflammasome and as a negative regulator of immune signaling (Tuladhar and Kanneganti, 2020). NLRP12-deficient mice are hyper-resistant to infection by *S. typhimurium* (Zaki et al., 2014). The expression of NLRP12 was lower in patients with UC compared to the other patient cohorts. Furthermore, mice with NLRP12 deficiency showed enhanced DSS-induced colitis and AOM/DSS-induced colon cancer due to dysbiosis and increased NF-κB signaling (Zaki et al., 2011; Allen et al., 2012; Chen et al., 2017). NLRP12 also played a role in HFD-induced obesity. HFD-fed Nlrp12^−/−^ mice showed increased weight, adipose deposition, and blood glucose levels compared to the wild-type mice; dysbiosis in the HFD-fed Nlrp12^−/−^ was marked by increased obesity-associated *Erysipelotrichaceae* and reduced *Lachnospiraceae* family and enzymes required for SCFA biosynthesis (Truax et al., 2018).

NLRC3 is a member of the NLR family that is significantly downregulated in the colorectal cancer tissues (Liu et al., 2015b). NLRC3 is an inhibitory nucleic acid sensor that is activated by the TLRs that attenuates type I IFN response by sequestering and inhibiting the stimulator of IFN genes (STING) protein (Schneider et al., 2012; Zhang et al., 2014b; Li et al., 2019c). NLRC3-deficient mice were highly susceptible to DSS-induced colitis and AOM/DSS-induced colorectal tumorigenesis. Mechanistically, NLRC3 inhibited activation of the mechanistic target of rapamycin kinase (mTOR) signaling pathway by blocking the activation of PI3K-dependent, which was triggered by engagement of the growth factor receptors or TLR4 (Karki et al., 2016).

### RLRs

RIG-I, melanoma differentiation-associated gene 5 (MDA5), and laboratory of genetics and physiology 2 (LGP2) are three major proteins belonging to the RIG-I-like receptor family. RIG-1 and MDA5 are PRRs with a RNA helicase domain and a CARD domain, whereas LGP2 contains only the RNA helicase domain and does not have a CARD domain (Moon and Stappenbeck, 2012; Brisse and Ly, 2019). RLRs are involved in viral infection. RIG-I binds to the 5ʹ-triphosphate RNA, whereas MDA5 recognizes long dsRNA structures. The recognition of viral RNA by the RLRs activated of the MAVS-dependent signaling pathway that results in the production of type I IFNs and ISGs (Li and Wu, 2021). LGP2 assisted MDA5–RNA interactions and enhanced MDA5-mediated antiviral signaling. LGP2 increased the initial rate of MDA5–RNA interactions and regulated MDA5 filament assembly, including the formation of numerous shorter MDA5 filaments with equivalent or higher activity than the longer filaments with only MDA5 (Bruns et al., 2014).

Both RIG-I and MDA5 are involved in the defense mechanisms against rotavirus infections in the intestine (Broquet et al., 2011). RIG-I and MDA5 are both upregulated in the IECs during rotavirus infection. Silencing of RIG-I, MDA5 or MAVS significantly decreased IFN-β production and increased rotavirus titers in the infected IECs. Rotavirus-infected mice with MAVS deficiency showed lower IFN-β levels, increased rotavirus titers in the IECs, and increased viral shedding in the feces. These results collectively demonstrated that the RIG-I/MDA5/MAVS signaling pathway played a significant role in protecting against viral infection in the intestine (Broquet et al., 2011). RIG-I in the antigen presenting cells (APCs) recognized the commensal viruses and maintained intestinal intraepithelial lymphocytes (IELs) through a type I IFN-independent, but MAVS/IRF1/IL-15 axis-dependent manner (Liu et al., 2019). Enteric bacteria and fungi are mostly linked to the IBD phenotypes. However, a recent study showed that perturbations in the intestinal virome or altered ability of RIG-I and MDA5 to sense the virome contributed to the induction of IBD (Adiliaghdam et al., 2022). IECs with the loss-of-function *MDA5* mutations (rs35744605, E627X) were associated with detrimental consequences for patients with IBD when exposed to viromes (Adiliaghdam et al., 2022). MAVS was downstream of RLRs and played a pivotal role in monitoring the commensal bacteria and preventing DSS-induced colitis by inducing multiple cytokines and antimicrobial peptides, including IFN-β and REG3γ (Li et al., 2011a). During acute intestinal tissue injury in mice, activation of RIG-I/MAVS or STING pathways induced IFN-I signaling and maintained integrity of the gut epithelial barrier and reduced the severity of GVHD (Fischer et al., 2017). Therefore, functional role of the RLR family members includes sensing the viral RNA, regulating the protective response against enteric virus infections, and maintaining intestinal homeostasis by sensing the commensal viruses.

### Other PRRs

Several other PRRs are also involved in modulating the host–microbiota interactions in the intestine. AIM2 is a cytosolic DNA sensor belonging to the ALR family. AIM2-deficient mice are highly susceptible to DSS-induced colitis and have a high colonic burden of *E. coli*, a commensal bacterium. The colonization of germ-free mice with microbiota from the AIM2-deficient mice caused higher susceptibility to colitis compared to the colonization of germ-free mice with microbiota from the wild-type mice. AIM2-deficient mice showed reduced production of IL-1β and IL-18, but infusion of IL-18 alleviated the higher colonic burden of *E*. *coli* and susceptibility to colitis (Hu et al., 2015). AIM2 sensed radiation-induced DNA damage in the nucleus and induced activation of the inflammasome and triggered cell death. This suggested that AIM2 was a promising therapeutic target for reducing the damage caused by exposure to ionizing radiations to tissues with actively proliferating cells such as the bone marrow and the gastrointestinal tract (Hu et al., 2016). Chemotherapeutic agent irinotecan (CPT-11) induced massive release of double-strand DNA from the intestine through exosome secretion. This dsDNA entered the cytosol of innate immune cells and activated the AIM2 inflammasome, which subsequently induced the synthesis and secretion of IL-1β and IL-18, both of which caused intestinal mucositis and late-onset diarrhea. The abrogation of AIM2 signaling, either in the AIM2-deficient mice or by a pharmacological inhibitor such as thalidomide, significantly reduced the incidence of drug-induced diarrhea without affecting the anti-cancer efficacy of CPT-11 (Lian et al., 2017). The expression of AIM2 is reduced in several cancer types. AIM2 suppressed colon tumorigenesis by interacting with the DNA-dependent protein kinase (Man et al., 2015; Wilson et al., 2015a). This function of AIM2 was independent of the inflammasome activation and IL-1β release. In the AOM/DSS and APC^Min^ models of CRC, *Aim2*-deficient mice showed higher tumor load than the control mice (Man et al., 2015; Wilson et al., 2015a). Therefore, AIM2 is a DNA sensor that responds to intestine damage and participates in colitis, pathogen invasion, and bowel cancer.

The cGAS-cGAMP-STING pathway is another cytosolic DNA-sensing pathway. In the presence of DNA, cGAS uses GTP and ATP to synthesize cyclic-di-GMP/AMP (cGAMP), which activated IFN signaling by binding with high affinity to STING (Brubaker et al., 2015). Alterations in the cGAS-STING DNA-sensing pathway adversely affected intestinal homeostasis but the underlying mechanisms are not clear. K63-linked ubiquitination of STING in the intestinal myeloid cells is elicited by the bacterial products including cGMP and the corresponding positive feedback loop drives intestinal inflammation (Shmuel-Galia et al., 2021). However, cGAS/STING-dependent IFN-β response triggered intestinal regeneration and recovery from radiation injury in the animal models (Leibowitz et al., 2021). In the colon cancer models under *Lactobacillus rhamnosus* GG therapy, the cGAS/STING-dependent type I IFN pathway improved the response to immune checkpoint blockade therapy (Si et al., 2022).

Dectin-1 is a member belonging to the CLR family that is expressed in the human IECs and human IEC lines, HT29 and SW480. Dectin-1 recognizes and responds to the fungal cell wall component β-glucan, and promotes chemokine secretion through phosphorylation of its downstream mediator Syk (Chan et al., 2009). Furthermore, β-glucan-induced chemokine secretion was not affected by the inhibition of v-raf-1 murine leukemia viral oncogene homolog 1 (RAF-1), which is involved in an alternate Dectin-1 responsive pathway. Therefore, it is critical to determine if Dectin-1 pathway in the IECs involves other downstream mediators such as CARD9/Bcl10/MALT1 (Cohen-Kedar et al., 2014). Mice lacking Dectin-1 exhibited increased susceptibility to chemically-induced colitis because of altered responses to indigenous fungi; *Dectin-1* gene polymorphism (CLEC7A) in humans is significantly associated with a severe form of UC (Iliev et al., 2012). Conversely, inhibition of Dectin-1 signaling ameliorated colitis by inducing *Lactobacillus*-mediated expansion of Treg cells in the intestine (Tang et al., 2015b). A recent study showed that deletion of both the Dectin-1 and Dectin-2 receptors significantly modified the bacterial microbiota but did not affect the fungal microbiota; moreover, these changes induced protective effects during colitis through members of the *Lachnospiraceae* family (Wang et al., 2022a). These conflicting results may be caused by the complex and variable microbiota populations and the gut environment. Specific intracellular adhesion molecule-3 grabbing non-integrin homolog-related 3 (SIGNR3), a mouse homolog of human DC-SIGN, belongs to the CLR family and recognizes fungi in the intestine (Eriksson et al., 2013). SIGNR3-deficient mice exhibited increased weight loss because of severe colitis symptoms compared to the wild-type littermates in a DSS-induced colitis model; SIGNR3-deficient mice showed increased inflammation in the colon with higher levels of TNF-α. Binding of the surface layer protein A derived from *Lactobacillus* to SIGNR3 exerted regulatory signals that mitigated colitis (Lightfoot et al., 2015).

### Metabolite-sensing receptors in the intestine

#### GPCRs

Intestinal GPCRs sense metabolites including SCFAs, medium-chain fatty acids (MCFAs), long-chain fatty acids (LCFAs), and others. SCFAs such as acetate, propionate, and butyrate are produced by the microbial fermentation of the dietary fiber and are the main energy sources for the colonocytes (Kaiko et al., 2016). SCFAs exert diverse effects on the functions of the mucosal immune cells and the IECs (Willemsen et al., 2003; Kaiko et al., 2016; Park et al., 2016; Zhao et al., 2018; Lavelle and Sokol, 2020). GPR43, GPR41, and GPR109a are the three known receptors for the SCFAs and are expressed on the IECs and the immune cells. SCFAs significantly increased the expression levels of GPR43 and GPR41 in the adipose tissue and reduced their expression in the colon (Lu et al., 2016). GPR43-deficient mice showed exacerbated or unresolved inflammation in the colitis models, thereby showing that stimulation of GPR43 by the SCFAs was necessary for the normal resolution of inflammatory responses under specific conditions (Maslowski et al., 2009). Furthermore, SCFA induced RegIIIγ and β-defensins in the intestinal epithelial enteroids from the wild-type mice but this was suppressed in the GPR43^−/−^ mice. This demonstrated that SCFAs promoted the synthesis of antimicrobial peptides in the IECs in a GPR43-dependent manner (Zhao et al., 2018). GPR109a, encoded by the *Niacr1* gene, is a receptor for butyrate and niacin, which are produced by the gut microbiota. GPR109a signaling promotes anti-inflammatory activity by inducing the differentiation of regulatory T cells (Tregs) and IL-10 production. Moreover, activation of GPR109a signaling by butyrate promoted IL-18 secretion in the colonic epithelium. *Niacr1*-deficient mice were highly susceptible to the development of colitis and colorectal tumorigenesis (Thangaraju et al., 2009; Singh et al., 2014). GPR109a also protected against colitis and facilitated dietary fiber-induced gut homeostasis by regulating the inflammasomes (Macia et al., 2015). GPR84 sensed MCFAs, which were derived from milk, especially coconut milk. GPR40 was also a sensor of MCFAs. GPR40 and GPR120 are involved in a spectrum of metabolic diseases and are sensors of LCFAs such as omega-3 fatty acids, which are derived mainly from fish (Tan et al., 2017; Mao et al., 2023). GPR35 can sense and bind products from tryptophan (Trp) and tyrosine catabolism. GPR91 binds succinate, an intermediate of the citric acid cycle. GPR119 is activated by three *N*-acylethanolamines (oleoylethanolamine, palmitoleoylethanolamine, and linoleylethanolamine) and 2-oleoylglycerol. Furthermore, linoleylethanolamine and 2-oleoylglycerol served as physiologically relevant endogenous GPR119 agonists that mediated receptor activation upon nutrient uptake (Syed et al., 2012). Lipid sensing in the enteroendocrine cells of the distal intestine by the fat receptor GPR119 regulates gut hormone levels, food intake, and body weight (Higuchi et al., 2020). Therefore, GPR119 plays a key role in metabolic functions. The G protein-coupled bile acid receptor (GP-BAR1, also named as TGR5) recognized secondary bile acids and was highly expressed in the ileum and the colon (Li and Chiang, 2014). Morphology of the colonic mucous cells and molecular architecture of the epithelial TJs were altered in the TGR5^−/−^ mice, thereby causing increased intestinal permeability and susceptibility to develop severe colitis in response to DSS (Cipriani et al., 2011). GPCRs consist of multiple orphan receptors for several ligands and have diverse functions. Therefore, further in-depth investigations are necessary in this area of research. Cohen and colleagues used bioinformatics and synthetic biology to mine the human microbiota and identify GPCR-interacting *N*-acyl amides. They reported that GPCRs targeted by the human microbial *N*-acyl amides were localized to the gastrointestinal tract and the associated immune cells (Cohen et al., 2015, 2017). In the mouse models, GPCRs were associated with diverse mucosal functions, including metabolism (GPR119, GPR120), immune cell differentiation (S1PR4, PTGIR, and PTGER4), immune cell trafficking (S1PR4, G2A) and tissue repair (PTGIR) (Cohen et al., 2017). Vmn2r26 is an olfactory receptor (a type of chemosensory GPCR) that is highly expressed on the Tuft-2 cells. It stimulated prostaglandin D2 (PGD2) production upon recognizing the *Shigella* metabolite *N*-undecanoylglycine (N-C11-G) and enhanced mucus secretion by the goblet cells in response to bacterial infections (Xiong et al., 2022). GPCRs also play a role in human UC. Inflamed UC regions are distinguished by the MAS related GPR family member X2 (MRGPRX2)-mediated activation of mast cells and decreased activation is observed in a UC-protective genetic variant of MRGPRX2 (Asn62Ser) (Chen et al., 2021b). Although several studies have characterized a whole plethora of GPCRs, the precise roles of each of these receptors are still evolving, especially in the immune responses (Tan et al., 2017).

#### AHRs

AHRs are ligand-inducible transcription factors that are expressed in the immune cells, epithelial cells, and few types of tumor cells. AHRs maintain homeostasis at the mucosal surfaces by recognizing xenobiotics, natural compounds such as Trp metabolites, and dietary components such as serotonin, melatonin, and vitamin B3 (Schiering et al., 2017). AhR expression was suppressed upon depletion of intestinal microbiota in the animal models and the intestinal tissue of patients with IBD, thereby suggesting a relationship between AhR expression and the gut microbiota (Monteleone et al., 2011; Wang et al., 2018a). In mice, deficiency of AhR in the IECs exacerbated inflammation in the DSS colitis model, but deletion of AhR in the T cells attenuated colitis and was manifested by the infiltration of Th17 cells into the lamina propria (Chinen et al., 2015). A recent study showed that mice with AHR repressor deficiency in the IELs were susceptible to infection with *Clostridium difficile* and DSS-induced colitis because excessive AHR signaling lead to oxidative stress and ferroptosis of the IELs and suppression of the intestinal immune responses (Panda et al., 2023). Highly adaptive *lactobacilli* produce indole-3-aldehyde, a Trp-indole derivative. Indole-3-aldehyde acted as an AHR ligand and contributed to AHR-dependent IL-22 transcription (Zelante et al., 2013; Agus et al., 2018). IL-22-dependent mucosal response resisted colonization of the fungus *Candida albicans*, protected the mucosa from inflammation, and was required for the survival of mixed microbial communities. Therefore, the microbiota–AHR axis represented an important strategy developed through co-evolutionary commensalism for fine-tuning host mucosal reactivity in accordance with Trp catabolism (Zelante et al., 2013; Stockinger et al., 2021). AHR signaling was also linked to intestinal inflammation through the downstream NF-κB-C/EBPβ signaling axis in the T cells and the DCs (Sanmarco et al., 2022). AHR signaling protected the stem cell niche and maintained the integrity of the intestinal barrier (Metidji et al., 2018). Besides, AHR played a key role in the regulation of IL-10R1 expression in the colon (Lanis et al., 2017). Dietary and microbiota-derived oxazole suppressed the production of IL-10 by the IECs and inflammation by activating AhR in the intestinal epithelium (Iyer et al., 2018). AhR expression in the gut also regulated the function and maintenance of the ILCs. Genetic or pharmacological activation of AhR suppressed ILC2 function but enhanced ILC3 maintenance to protect the host from *Citrobacter rodentium* infection (Li et al., 2018). Furthermore, AHR signaling in the enteric neurons acted as a regulatory node to maintain gut homeostasis and health; neuron-specific deletion of AhR reduced peristaltic activity of the colon; expression of AhR in the enteric neurons of mice partially restored intestinal motility when treated with antibiotics (Obata et al., 2020). In summary, AhR was expressed widely in the gut and sensed several metabolites, especially derivatives of Trp to regulate the function of T cells, IECs, and ILCs, and maintain gut homeostasis.

#### Nuclear receptors

Nuclear receptors are a group of ligand-activated transcription factors that play important roles in embryogenesis, development, and metabolism (Li and Chiang, 2014). Farnesoid X receptor (FXR), Pregnane X receptor (PXR), and Vitamin D receptor (VDR) are three nuclear receptors that directly bind to the bile acids, which are small molecules with a steroidal structure and are synthesized from cholesterol by the liver hepatocytes. Primary bile acids are secreted into the small intestine, reabsorbed in the terminal ileum, transported back to the liver via portal circulation, and further metabolized by the gut microbiota to generate secondary bile acids. Bile acids facilitate intestinal digestion and absorption of dietary fat, steroids, drugs, and lipophilic vitamins. FXR and PXR are highly expressed in the tissues exposed to the bile acids such as the liver and the intestine, whereas VDR is widely expressed in most tissues (Li and Chiang, 2014). Both primary and secondary bile acids activated FXR signaling, which regulates bile acid synthesis, metabolism, and intake by the host. Mice lacking FXR showed increased levels of bacteria in the ileum and compromised integrity of the epithelial barrier (Inagaki et al., 2006). FXR activation inhibited inflammation, promoted the synthesis of cathelicidin, an antimicrobial peptide (D’Aldebert et al., 2009), and protected the integrity of the intestinal barrier in IBD (Gadaleta et al., 2011; Eshleman and Alenghat, 2021; Chen et al., 2022). The extracts of *Citrus aurantium L*. exerted protective effects by modulating the FXR/fibroblast growth factor 15 (FGF15) pathway and the FXR-targeted proteins and restored the composition of the intestinal microbiota, reshaped the barrier integrity, and maintained homeostasis of the bile acids (Liu et al., 2020). Ursodeoxycholic acid (UDCA) is an off-patent drug that reduced ACE2 levels by inhibiting FXR signaling in the lungs, cholangiocytes, and intestinal organoids of humans, mice, and hamsters, thereby suggesting that FXR inhibition may protect against SARS-CoV-2 infection (Brevini et al., 2023a). In the pluripotent stem cell (PSC)-derived hepatocyte like cells (HLCs), which demonstrated a liver-intestine hybrid state, combined FXR expression plus agonist exposure enhanced the expression of hepatocyte-associated genes and increased the ability of bile canalicular secretion as well as the lipid droplet formation (Nell et al., 2022). These data demonstrate that FXR signaling played a significant role in the metabolic, gastrointestinal, and liver diseases, but the exact details require further investigation (Sun et al., 2021). Several studies have shown that vitamin D-VDR signaling promotes innate immune responses (Ismailova and White, 2022). The downstream VDR target genes were upregulated in the colon tissues of the HFD-fed mice; moreover, combinatorial treatment of the intestinal HT29 epithelial cells with lipids and bile acids, and modulation of the vitamin D targeting pathways protected against colitis and colitis-associated cancer risk (O’Mahony et al., 2023).

These data showed that PRRs and other metabolite receptors played pivotal roles in maintaining gut homeostasis by sensing the intestinal microbes and the dietary and microbiota-derived metabolites.

In summary, the microbial sensing receptors influence intestinal homeostasis through several mechanisms. Firstly, the PRRs sense foreign pathogens and activate downstream signaling mechanisms to promote synthesis of the pro-inflammatory cytokines, which eventually protect against pathogen evasion and maintain the gut homeostasis. Secondly, the highly mutualistic relationship between the host and the gut microbiota is partly mediated through PRR signaling. Thirdly, microbiota-derived metabolites regulated the differentiation and functions of host intestinal cells through metabolite sensor signaling pathways. Although significant data has been generated to understand the interactions between the sensors and the microbiota, several questions remain that require further investigations.

## Intestinal cells in the host–microbiota interactions

**Figure 2.**
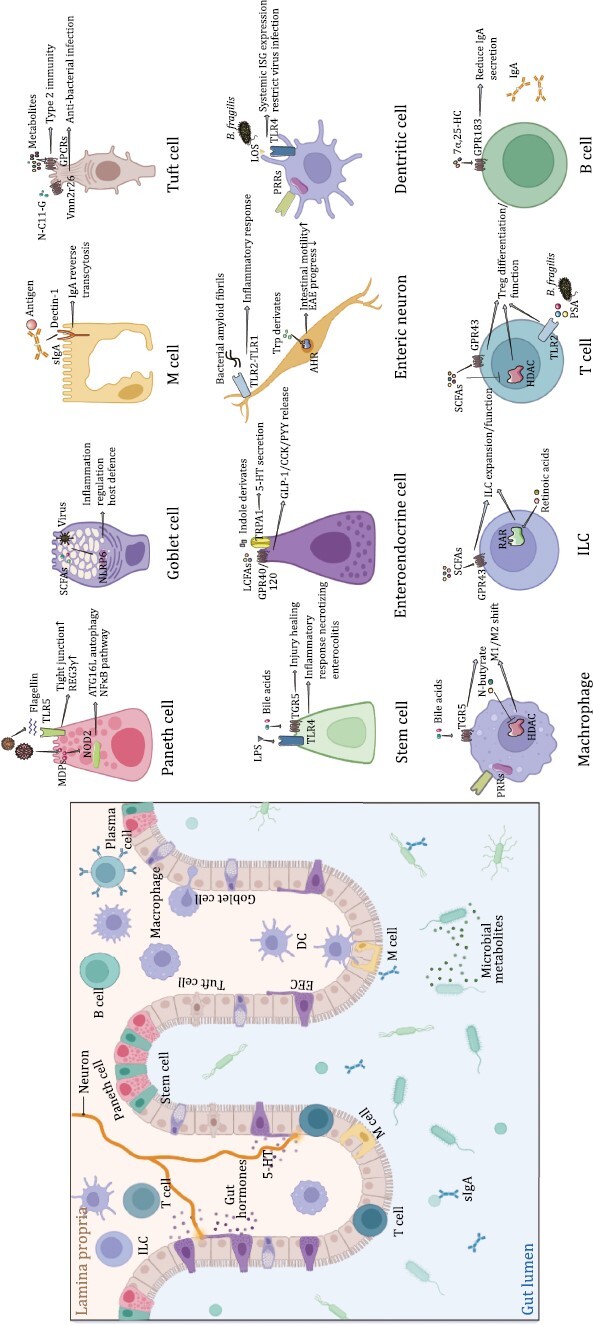
**Ligands and functions of microbial sensors in different cell types in the intestine.** The diagram on the left depicts the distribution of different intestinal epithelial cells (IECs) and immune cell types in the intestine, and the diagram on the right elaborates several typical signaling pathways through which specific cell types from the intestine take function. Paneth cell expresses NOD2 and TLR5 respectively to respond to bacterial MDP and flagellin. Genetically, Nod2 is highly associated with inflammatory bowel disease (IBD). NLRP6 is important for goblet cell to prime inflammatory responses, defend virus infection and mucus secretion. Dectin-1 is required for the reverse endocytosis of the secretory IgA (sIgA)–antigen complex in M cells. Tuft cells would express lots of GPCRs to recognize different metabolites, such as Tuft-2 cells express orphan receptor Vmn2r26 to recognize metabolite *N*-undecanoylglycine (N-C11-G). LPS–TLR4 interaction help intestinal stem cells to induce inflammatory responses and bile acids–TGR5 interaction is important for stem cell proliferation to proceed wound healing. Enteroendocrine cell (EEC) could also be considered as nervous cell and is important in producing gut hormones. TRPA1 and GPR40/120 expressed in EECs could be activated by corresponding ligands and induce 5-Hydroxytryptamine (5-HT) or glucagon-like peptide-1 (GLP-1)/cholecystokinin (CCK)/peptide tyrosine tyrosine (PYY) release. Recognition of microbial signals occurring on enteric neurons is also important for host health. Bacteria can release amyloid protein to stimulate TLR2–TLR1 dimers on neurons and induce neuroinflammation. Meanwhile, tryptophan derivatives released by bacteria can also activate AHR in the nucleus of neurons and increase intestinal motility. As classic antigen presenting cells, myeloid cells including dendritic cells (DCs) and macrophages play an important role in host immune response and tolerance formation. They both express a lot of PRRs for microbial recognition. Symbiotic microorganisms such as *Bacteroides fragilis* regulate natural resistance to viral infection by inducing IFN-I via intestinal DCs through its outer membrane component lipooligosaccharide (LOS) associated polysaccharide A (PSA). TGR5 signaling and HDAC activation regulated by microbial metabolites could modulate macrophage M1/M2 polarization shift. ILC3s expressed GPR43 in colon to sense SCFAs, and take retinoic acid (RA)-RAR signaling in SI to promote cell expansion. Lymphocytes have always been the important members of the intestinal immune system. For Treg cells, SCFAs signal through GPR43 or HDAC are both important to maintain Treg population and function. Furthermore, PSA signals from *B. fragilis* directly activate TLR2 on Treg cells to promote induced Treg (iTreg) cell differentiation. With the participation of MyD88-dependent intestinal flora recognition, IECs absorb dietary cholesterol to produce 7α,25-dihydroxycholesterol (7α,25-HC). 7α,25-HC can be then sensed by plasma B cells via chemotactic receptor GPR183 and finally inhibits antigen-specific IgA secretion.

Host immune cells in the intestine maintain intestinal homeostasis by sensing the microbial signals. The intestinal immune cells exert an “inside-out” control to determine the microbiota localization and composition of the microbial community. At the same time, microbiota exerts an “outside-in” effect on the intestinal immune cell activity and development. In this section, we will discuss interactions between the gut microbiota and the immune cells that govern innate or adaptive immune responses (Fig. 2).

### Classical and non-classical innate immune cells

Normal functioning of the innate immune system is required to maintain healthy gut microbiota. Dysbiosis causes inflammation because innate immune cells are aberrantly activated. The innate immune cells are the first defense barrier against the foreign agents. Therefore, they maintain gut homeostasis by expressing significantly higher levels of PRRs and other microbial sensors and act as effective sensing and signaling units.

#### IECs

IECs function as a physical barrier between the host’s internal milieu and the gut luminal environment. Therefore, the barrier function is impaired because of defects in the TJ components such as ZO-1, Claudin-4, and Occludin between the IECs. This causes increased exposure to the gut microbiota and uptake of the luminal antigens, and leads to aberrant activation of the mucosal immune system (Cario et al., 2007; Hartmann et al., 2012). Urolithin A is a microbial metabolite derived from the polyphenols that are naturally found in fruits such as berries and pomegranate. Urolithin A and its potent synthetic analogue, UAS03, protect against colon diseases by enhancing barrier functions and reducing inflammation through upregulation of epithelial TJ proteins via activation of AhR-nuclear factor erythroid 2-related factor 2 (NRF2)-dependent pathways (Singh et al., 2019a). Furthermore, the physical barrier is reinforced by various biochemical adaptations. For example, IECs secrete a broad range of antimicrobial peptides, including defensins, cathelicidins, and calprotectins (Ganz, 2003; Salzman et al., 2003). These diverse group of peptides confer broad-spectrum antimicrobial properties by forming pores in the bacterial cell wall and obstruct the entry of commensal and pathogenic bacteria into the underlying lamina propria (Artis, 2008). PRR signaling represents one of the mechanisms through which IECs respond to the microbes in the lumen. Furthermore, IECs utilize various mechanisms to regulate PRR signaling and optimize immune responses. For example, Single Ig IL-1 Related Receptor (SIGIRR), a negative regulator of IL-1 and TLR signaling, is responsible for the hypo-responsiveness of the IECs and promotes resistance of the commensal microbiota against the bacterial pathogens (Sham et al., 2013). Therefore, IECs acquire TLR tolerance immediately after birth by exposure to exogenous endotoxins to facilitate microbial colonization through posttranscriptional down-regulation of the interleukin 1 receptor-associated kinase 1, which is essential for the *in vitro* epithelial TLR4 signaling (Lotz et al., 2006).

IECs play an integral role in the discrimination between pathogenic and commensal bacteria and the subsequent regulation of immune responses in the intestinal microenvironment. IECs include a wide variety of cells such as the enterocytes, stem cells, EECs, Paneth cells, goblet cells, M cells, and Tuft cells, which express a wide range of PRRs that are activated upon recognizing the microbial components and maintain intestinal homeostasis by activating immune responses (Pott and Hornef, 2012). The niche of ISCs are at the forefront of host-microbe activity and are critical for regulating nutrient absorption, endocrine signaling, energy homeostasis, immune responses, and systemic health (Peck et al., 2017). TGR5 senses bile acids and promotes renewal of ISCs to drive regeneration in response to injury (Sorrentino et al., 2020). Paneth cells express elevated levels of NOD2, which are activated by the microbial peptidoglycans. These induce pro-inflammatory cytokines, autophagy, and epithelial regeneration, which in turn regulate the composition of the gut microbiota (Couturier-Maillard et al., 2013; Nigro et al., 2014; Ramanan et al., 2014). NLRP6-dependent mechanisms contribute to the secretion of mucus by the goblet cells. NLRP6 deficiency significantly reduced mucus secretion in the large intestinal lumen because of defective autophagy in the goblet cells. This caused dysbiosis and hyper-susceptibility to enteric infections (Elinav et al., 2011; Wlodarska et al., 2014; Levy et al., 2015; Birchenough et al., 2016). A subset of Trp-derived indole derivatives produced by *Edwardsiella tarda* directly stimulate epithelial sensory EECs through the human and mouse receptor transient receptor potential ankyrin A1 (TRPA1); moreover, intestinal 5-Hydroxytryptamine (5-HT) secretion by the activated Trpa1^+^ EECs regulated the enteric and vagal neuronal pathways (Ye et al., 2021). Tuft cells orchestrated antiparasitic immunity in the gut by acting as sentinels of type 2 immunity against the intestinal parasites (Howitt et al., 2016). Tuft cells triggered type 2 immunity by expressing a myriad of GPCRs, which sensed microbes and metabolites such as succinate that are derived from the host, microbes, and diet (Nadjsombati et al., 2018; O’Leary et al., 2019; Banerjee et al., 2020). CD45^+^ Tuft-2 cells quickly expand in response to the bacterial infections by sensing the bacterial metabolite *N*-undecanoylglycine through the vomeronasal receptor Vmn2r26 (Xiong et al., 2022). ALK2, a bone morphogenetic protein (BMP) receptor, played a key role in the negative feedback loop that regulated immune type 2-driven tuft cell hyperplasia (Lindholm et al., 2022). M cells are highly specialized epithelial cells of the gut-associated lymphoid tissue (GALT). They take up intestinal microbial antigens, transport them from the gut lumen to the underlying immune system, and stimulate an efficient mucosal immune response. M cells sense and bind to the secretory IgA-antigen complex through Dectin-1. Then, the bound secretory IgA-antigen complex is transferred and internalized via DC-SIGN by the DCs, which induce the mucosal immune responses (Rochereau et al., 2013). NOD2 modulated SIgA transport through the human and mouse M cells by downregulating the expression of two retrograde transport receptors, Dectin-1 and Siglec-5 (Rochereau et al., 2021). Transmembrane mucins were expressed on the apical surface of the enterocytes and participated in the terminal digestion of polysaccharides and peptides as well as absorption of various nutrients. The dysfunction or damage of enterocytes cause intestinal diseases. *In vivo* infection with *E*. *coli* induced the formation of phosphorylated H2AX foci in the mouse colon enterocytes and contributed to the development of sporadic colorectal cancer (Cuevas-Ramos et al., 2010). Thus, IECs serve not only as a mucosal barrier but also discriminate between the pathogens and the commensals.

#### Myeloid cells

Myeloid cells located in the lamina propria play a significant role in the defense against pathogenic antigens and perform a regulatory role in the immune homeostasis. The commensal-bearing DCs induce protective secretory IgAs, which are distributed throughout all the mucosal surfaces through recirculation of the activated B and T cells (Macpherson and Uhr, 2004). Gut macrophages develop a unique phenotype called as “inflammation anergy”, which is characterized by the induction of a non-inflammatory profile when the intestinal macrophages encounter microbial stimuli under homeostatic conditions (Smythies et al., 2005). Macrophages and DCs are critical APCs that require PRR signaling for maturation and efficient antigen presentation and T cell activation. Therefore, they play a crucial role in the crosstalk between innate and adaptive immune responses (Geremia et al., 2014; Spindler et al., 2022).

Microbiota composition affects the development and function of the myeloid cells. For example, *in vivo* aging analysis in mice showed that neutrophil y was driven by the microbiota through TLR- and MyD88-dependent signaling pathways. Furthermore, depletion of the microbiota reduced the number of circulating aged neutrophils and improved the pathogenesis and inflammation-related organ damage in the mouse models of endotoxin-induced septic shock and sickle cell disease (Zhang et al., 2015). Bacterial strains such as *E. faecalis*, *Streptococcus gallolyticus*, and *E*. *coli* induced differentiation and function of the colon-resident macrophages (Wang and Huycke, 2007; Raisch et al., 2015). *Fusobacterium nucleatum* induced immunosuppression by triggering M2 macrophage polarization through TLR4 and IL-6/STAT3/c-MYC signaling, thereby enhancing colorectal tumor growth (Chen et al., 2018). Intestinal muscularis macrophages responded to luminal infection by upregulating a neuroprotective program through β2-adrenergic receptor signaling, and limited enteric-associated neuronal damage through the arginase 1-polyamine axis (Matheis et al., 2020). Gut microbiota-derived metabolites also orchestrated functions of the resident macrophages (Levy et al., 2016). Stimulation of GPR109a signaling by niacin or butyrate promoted anti-inflammatory properties of the colon-resident macrophages, which then induced differentiation of the Treg cells and the IL-10-producing T cells (Singh et al., 2014). *N*-butyrate regulated selective functions of the lamina propria macrophages and maintained intestinal homeostasis by directly inhibiting the histone deacetylases (Chang et al., 2014; Schulthess et al., 2019). Tryptamine and indole-3-acetate reduced fatty acid- and LPS-stimulated production of pro-inflammatory cytokines by the macrophages and inhibited the migration of macrophages towards the chemokine in an AhR-dependent manner (Krishnan et al., 2018). TGR5 is a G protein-couple receptor for secondary bile acids expressed on the circulating monocytes and the colon-resident macrophages. It promoted an IL-10- and age-dependent shift towards the alternative M1/M2 macrophage phenotypes (Biagioli et al., 2017; Becker et al., 2019). Putrescine is a polyamine that functions as a substrate for symbiotic metabolism. It increased the abundance of anti-inflammatory macrophages in the colon and ameliorated the symptoms of DSS-induced colitis in mice (Nakamura et al., 2021). Furthermore, germ-free animals showed extensive defects in the development of intestinal innate immune cells. This indicated that microbiota was essential for the normal development and functioning of the host innate immune system. The number of intestinal macrophages was normal in the germ-free mice, but the functions of peritoneal macrophages including chemotaxis, phagocytosis and antimicrobial activity were aberrant (Morland and Midtvedt, 1984; Williams et al., 2006). The number of DCs was also reduced in the intestines of the germ-free mice, but mono-colonization with *E*. *coli* was sufficient to recruit the DCs into the intestinal tissues of the germ-free mice (Williams et al., 2006). Intestinal DCs established oral tolerance against antigens derived from the food, commensal microbiota, and metabolites while initiating immune responses against the mucosal pathogens in the intestine (Sun et al., 2020). The activation of PRRs such as TLRs promoted the maturation of DCs with upregulation of the costimulatory molecules and secretion of the pro-inflammatory cytokines (Iwasaki and Medzhitov, 2010; Schenten and Medzhitov, 2011). And these days, several studies proved that intestinal microbiome could restrict virus infection through activating PRRs in gut DCs followed by downstream type I IFN release which could induce systemic ISGs expression and natural virus resistance (Stefan et al., 2020; Winkler et al., 2020). DCs also used the lysosomal histidine/peptide solute carrier transporter SLC15A4 to couple phagocytosis (the necessary first step to sense foreign antigens) with NLRC4 inflammasome assembly and activity (López-Haber et al., 2022).

#### ILCs

ILCs constitute a newly identified lymphoid lineage that protects the intestine from pathogen invasion by contributing to development of the gut lymphoid tissue and cooperating with the IECs and other innate and adaptive immune cells to repair gut injury (Bostick and Zhou, 2016). ILCs are classified into three main groups based on the expression of specific transcriptional factors: (i) T-bet^+^ ILC1s (ILC1s and natural killer (NK) cells); (ii) GATA binding protein 3 (GATA3^+^) ILC2s; and (iii) retinoic acid receptor-related orphan receptor-γt (RORγt^+^) ILC3s (ILC3s and lymphoid tissue-inducer (LTi) cells) (Spits and Di Santo, 2011; Walker et al., 2013). ILCs are mainly located at the epithelial surfaces and sense nutrients, environmental xenobiotics, and microbiota through the AhR, Retinoic acid receptors (RARs), retinoid X receptors (RXRs), TLRs, and other cell surface receptors (Kiss et al., 2011; Sawa et al., 2011; Spencer et al., 2014; van de Pavert et al., 2014). The genomic landscape of the intestinal ILCs has been characterized through integrated genome-wide RNA-seq, ChIP-seq, ATAC-seq, and single-cell transcriptome analyses (Gury-BenAri et al., 2016). The immune function of the ILC3s is most well-studied among the ILCs in the intestine. ILC3s are comprised of a heterogeneous population of cells that protect against bacterial and fungal invasion, and regulate the commensal microbiota populations and lymphoid tissue development. Activated ILC3s produce IL-17A, IL-22, TNF, and GM-CSF (Takatori et al., 2009; Mortha et al., 2014). GPR43 expressed on the colon-resident ILC3s senses SCFAs and promotes expansion and function of the ILC3s; however, in the small intestine, retinoic acid signaling promotes expansion and function of the ILC3s (Chun et al., 2019). Furthermore, microbiota-derived SCFAs regulated the optimal numbers of ILCs and promoted IL-22 production in the ILCs through GPCRs and AhRs by inhibiting the histone deacetylase (Yang et al., 2020; Sepahi et al., 2021). ILC3s also produce soluble lymphotoxin-α, which regulate T cell-dependent IgA production in the lamina propria (Kruglov et al., 2013). ILC populations and functions play an important role in maintaining intestinal homeostasis and during inflammatory conditions such as IBD (Saez et al., 2021; Coman et al., 2022).

### Adaptive immune cells

RAG1-deficient mice lacking adaptive immunity showed alterations in their microbiota populations (Kato et al., 2014). Furthermore, germ-free mice showed extensive defects in the development of GALT (Round and Mazmanian, 2009). This suggested a critical link between adaptive immunity and the gut microbiota. Although the role of T cells or B cells in microbial sensing is not discussed generally, several molecules belonging to the microbial sensing pathways are expressed in the adaptive immune cells and serve important functions (Kabelitz, 2007). Extensive studies have shown that the antigen-specific adaptive immune responses influence the mutualistic relationship between host and gut microbiota.

#### T cells

The complicated environmental factors and the unique structure of the mammalian intestine influence the distribution of a wide range of T lymphocyte subsets in the IECs and their scattered distribution in the lamina propria. These intestine-resident T cells show distinct effector functions and play a pivotal role in maintaining the gut homeostasis. The innate antigen presenting cells play a significant role in the function, differentiation, and proliferation of the intestinal T cells. For example, microbiota-induced intestinal RORγt^+^ Tregs depend on the DCs and the major histocompatibility complex (MHC) class II to differentiate into a specific subset of Th17 cells (Ohnmacht et al., 2015). TLRs are primary sensors of the microbiota and are expressed on both the innate and the adaptive immune systems. T cell activation and functions (Kabelitz, 2007) are regulated by TLR signaling and TLR components such as TLR2 (Liu et al., 2006; Sutmuller et al., 2006; Wang et al., 2006; Chen et al., 2009), TLR3 (Wesch et al., 2006), TLR4 (Caramalho et al., 2003), TLR8 (Peng et al., 2005), MyD88 (Gelman et al., 2006; Fukata et al., 2008; Geuking et al., 2011), TRIF (Geuking et al., 2011), and other PRRs regulating TLR signaling (Negishi et al., 2012).

Treg cells play an important role in maintaining immune tolerance to the gut microbiota and the dietary antigens (Round and Mazmanian, 2010; Geuking et al., 2011; Bilate and Lafaille, 2012). Both thymus-derived Tregs (tTregs) and peripherally differentiated Tregs (pTregs) are found in the intestine (Ohnmacht et al., 2015; Sefik et al., 2015). Germ-free mice lack pTregs. This suggested that the pTregs were induced and maintained by the microbiota. Furthermore, in-depth investigation of the fate of immature T cells expressing the transgenic T cell receptor (TCR) showed that the differentiation of Treg cells did not occur in the thymus but occurred in the colon after exposure to the microbiota (Lathrop et al., 2011). Tregs in the intestine use the TLRs to directly sense the microbes. Polysaccharide A derived from a commensal bacterium *B. fragilis* signaled directly through TLR2 on the Foxp3^+^ Treg cells and promotes iTreg cell differentiation (Caesar et al., 2015; Cao et al., 2015) and suppressed immunity by inhibiting the Th17 cell responses (Round et al., 2011).

T cell-specific MyD88 signaling is required for the induction of both Th1 and Th17 responses after overcoming Treg suppression through IL-1 signaling (Schenten et al., 2014). Treg-specific Myd88 deletion mice showed deficiency in the intestinal Treg cells, increased numbers of TH17 cells, and enhanced IL-17-dependent inflammation in experimental colitis (Wang et al., 2015b). This demonstrated that TLR-MyD88 signaling in the Treg cells promoted mucosal tolerance by inducing the anti-commensal IgA-responses (Wang et al., 2015b). Experiments with the *Clostridiales* species-associated food allergy model showed that the commensal microbiota activated the MyD88–RORγt pathway in the nascent Treg cells to mediate the effects of bacteriotherapy against food allergy and this mechanisms included restoring the RORγt^+^ Treg population and re-establishing tolerance; however, dysbiosis impaired this Treg cell response (Abdel-Gadir et al., 2019). These data demonstrated that MyD88 was indispensable for mucosal T cell induction and activities.

Other PRR signaling pathways also exhibit regulatory functions in the gut T cells. For example, transfer of the CD4^+^CD45RB^hi^Nlrp12^−/−^ T cells into the immunodeficient mice exacerbated severe colitis and showed that NLRP12 was an intrinsic negative regulator of T cell activation (Lukens et al., 2015). NLRC3 attenuated IFN-γ and TNF expression in the CD4^+^ T cells and reduced Th1 and Th17 cell proliferation in a T cell-intrinsic manner (Uchimura et al., 2018). The cytosolic DNA sensor AIM2 also showed an intrinsic role in the T cells for promoting the stability of Treg cells during inflammation. AIM2 was highly expressed by both the human and mouse Treg cells. AIM2 altered metabolism and enhanced the stability of Treg cells by reducing AKT-mTOR signaling (Chou et al., 2021). These data demonstrated a novel role for the PRRs in the T cell functions.

Th17 cell differentiation was reduced and Treg cell differentiation was increased by the microbiota-derived metabolites, including bile acids. Furthermore, SCFAs enhanced the differentiation of Treg cells in the intestinal lamina propria (Furusawa et al., 2013; Hang et al., 2019). SCFAs influenced the activity and differentiation of the peripheral T cells, especially Tregs, by inhibiting HDACs. In mice, the production of butyrate by the commensal microorganisms during starch fermentation facilitated extrathymic generation of the Treg cells. Propionate was another SCFA of microbial origin that regulated *de novo* generation of peripheral Tregs through HDAC inhibition. However, acetate, another SCFA generated by the microbiota, lacked HDAC-inhibitory activity. SCFAs transmitted signals through the GPCRs. For example, SCFAs regulated the size and function of the colon-resident Tregs and protected against colitis in a GPR43-dependent manner (Smith et al., 2013). These results demonstrated that the bacterial metabolites mediated the communication between the commensal microbiota and the immune system and influenced the fate of gut-resident T cells (Arpaia et al., 2013). However, several studies suggested that SCFAs did not induce the RORγt^+^ Treg cells effectively (Sefik et al., 2015; Abdel-Gadir et al., 2019). Trp derivate indole-3-aldehyde generated by the *Lactobacillus reuteri* D8 stimulated the LPLs through AhR to secrete IL-22 and induced recovery of the damaged intestinal mucosa by accelerating the proliferation of IECs through increased STAT3 phosphorylation (Hou et al., 2018). Fatty acid sensor GPR120 regulated IL-10 expression in the intestinal CD4^+^ T cells and suppressed DSS-induced colitis development in mice (Yang et al., 2022). In the CD patients, treatment with oral vitamin D reduced VDR expression in the CD4^+^ T cells (Bendix et al., 2015). However, in mice, VDR binding to NLRP3 restricted IL-4 expression and prevented biased Th2 polarization (Bendix et al., 2015; Huang et al., 2018). Bacterial metabolites in the gut–liver axis and the tissue stromal factors regulated liver immunity by driving the myeloid instruction of CD8^+^ T cells with immunomodulatory ability (Pallett et al., 2023). These data demonstrated that the microbiota-derived metabolites significantly modulated immune function by directly regulating signaling in the T cells. Conversely, acute SFB colonization stimulated Th17 cells, which then initiated a dynamic IL-17A-CXCR2-neutrophil axis that limited microbiota colonization (Flannigan et al., 2017).

#### B cells

Intestinal B cell activation and differentiation is highly dependent on the gut microbiota. In turn, B cells regulated the gut microbiota and maintained intestinal homeostasis, through the production of immunoglobulins (Igs). IgA is the most abundant antibody isotype that is secreted into the gut lumen. It binds to the gut microbiota and prevents their direct interaction with the host. The function of IgA in shaping the microbiota was first reported in mice that are deficient in the activation-induced cytidine deaminase (AID), which is critical for antibody isotype switching. Mice lacking AID showed hyperplasia of the ILFs and a 100-fold expansion of the anaerobic commensal bacteria in the intestine (Fagarasan et al., 2002). SIgA binds or “coats” microbes and prevents direct interaction between the microbes or other antigens and the host via immune exclusion (Yang and Palm, 2020). The coating of commensal microbes such as *Lactobacillus*, *Akkermansia* and some colitogenic bacteria with sIgA was higher in the healthy humans and mice compared to those with malnutrition or colitis (Palm et al., 2014; Kau et al., 2015; Huus et al., 2020). Furthermore, MyD88-mediated signaling was required for the development of intestinal IgA^+^ B cells. Loss of MyD88-mediated signaling diminished targeting of the gut microbiota by high-affinity IgA and resulted in a failure to regulate the growth and homeostasis of the bacterial communities (Kubinak et al., 2015). TLR5-deficient mice showed reduced levels of specific anti-flagellin IgA and contributed to the overexpression of flagellar genes by the commensal microbiota (Cullender et al., 2013). A subset of TLR5^+^CD11c^hi^CD11b^hi^ DCs in the lamina propria induced the differentiation of naive B cells into IgA-producing plasma cells in the intestine (Uematsu et al., 2008). The absorption of dietary cholesterol and the recognition of commensal bacteria by the duodenal IECs stimulated the production of oxysterols such as 7α,25-dihydroxycholesterol, which are evolutionarily conserved lipids that suppress IgA secretion by the plasma cells through the GPR183 receptor. This suggested that B cells were regulated by the dietary metabolites (Ceglia et al., 2023). IgA production by the B cells was critical for maintaining a diverse and healthy composition of the gut microbiota. Diverse and selective IgA secretion in the gut reduced microbiota complexity in the T cell-deficient mice caused gut mucosal dysbiosis (Kawamoto et al., 2014). Murein lipoprotein is a highly conserved outer membrane protein of the Gram-negative bacteria and is a major antigen that induces TLR4-dependent IgG production in both mice and humans (Zeng et al., 2016). Multiple studies have demonstrated that the presence of IL-10–producing regulatory B cells in humans prevents chronic colitis. Resident enteric bacteria stimulate the intestinal regulatory B cells through the TLR2 signaling pathways to produce IL-10, which in turn suppresses colonic T cell activation and maintains mucosal homeostasis (Mishima et al., 2019). In conclusion, B cells regulate homeostasis of both the immune responses and the gut microbiota by secreting sIgA and through other cellular sensors.

In summary, the gut microbiota sensing pathways have direct consequences on the regulation of barrier-forming epithelial cells, APCs, cytokine-secreting T cells including T helper cells, and the IgA-producing B cells in the gut. The signals from the local environment in the intestinal tract act on the innate immune cells and control the differentiation, migration, and maintenance of the adaptive immune cells. These cells maintain immune homeostasis by suppressing the host immune responses against harmless antigens and enforcing the integrity and functioning of the gut mucosal barrier.

## Microbial sensing in health and disease

Symbiotic relationship between the gut microbiota and the host has co-evolved over millions of years and is mutually beneficial. This mutualistic relationship is critical for intestinal homeostasis and overall human health. Therefore, dysbiosis caused by decreased or increased intestinal microbial signaling plays a central role in several human diseases, including infections, metabolic syndrome, cancer, neurological disorders, and autoimmune disorders (Tlaskalova-Hogenova et al., 2011). Dysbiosis causes failure of immunological tolerance, persistent inflammation, and immune regulation of remote organs, all of which contributes to the initiation and progression of these diseases (Tables 1 and 2).

**Table 1.** Sensors of microbial components in the intestine.

	Expression	Microbial signals	Diseases associated	
TLR	–	–	–	
TLR2	Enterocyte, GC, CSC, PC (Xiao et al., 2019); ENS; MC; TC; BC	LTA, peptidoglycan, lipoarabinomannan of bacteria; Lipopeptide/Lipoprotein of bacteria; Porin PorB of Neisseri (Reiser et al., 2017); Zymosan of fugus (Kataoka et al., 2002)	Mouse model	
			Colitis	Watanabe et al. (2006), Podolsky et al. (2009), Brun et al. (2013), Scheeren et al. (2014), Kashiwagi et al. (2015), Mishima et al. (2019) and Jia et al. (2022)
			Neurological disorders	Brun et al. (2013) and Oppong et al. (2013)
			CRC	Scheeren et al. (2014) and Tsoi et al. (2017)
			Metabolic syndrome	Plovier et al. (2017) and Miani et al. (2018)
			Human study	
			IBD	Pierik et al. (2006)
			PD	Gorecki et al. (2020)
TLR3	Enterocyte; MC; TC	dsRNA of virus	Mouse model	
			Colitis	Kawashima et al. (2013) and Yang et al. (2016)
			Human study	
			IBD	Cario and Podolsky (2000) and Yang et al. (2016)
			CRC	Castro et al. (2011)
TLR4	Enterocyte, GC, CSC; ENS; MC; TC; BC	LPS of Gram-negative bacteria	Mouse model	
			Colitis	Fukata et al. (2005), Shi et al. (2006a), Chaniotou et al. (2010) and Yang et al. (2017)
			CRC	Kuo et al. (2015), Tsoi et al. (2017), Li et al. (2019b), Xiang et al. (2020) and Burgueño et al. (2021)
			Metabolic syndrome	Giles et al. (2017) and Chen et al. (2021a)
			Neurological disorders	Perez-Pardo et al. (2019)
			Human study	
			PD	Zhao et al. (2015), Li et al. (2019d) and Perez-Pardo et al. (2019)
			CRC	Li et al. (2014)
TLR5	PC, CSC; DC	Flagellin of bacteria	Mouse model	
			Metabolic syndrome	Vijay-Kumar et al. (2010), Chassaing et al. (2014), Singh et al. (2015) and Scheithauer et al. (2022)
			Rotavirus infection	Vijay-Kumar et al. (2008b) and Zhang et al. (2014a)
			Colitis	Lodes et al. (2004), Vijay-Kumar et al. (2007), Carvalho et al. (2012a, 2012c) and Chassaing et al. (2014)
			Human study	
			IBD	Lodes et al. (2004)
			CRC	Beilmann-Lehtonen et al. (2021)
TLR7	Enterocyte; DC	ssRNA of virus	Mouse model	
			Colitis	Sainathan et al. (2012), Yang et al. (2016) and McAlpine et al. (2018)
			Human study	
			IBD	Yang et al. (2016)
TLR9	Enterocyte; ENS; MC	Non-methylated CpG DNA of bacteria and virus	Mouse model	
			Colitis	Rachmilewitz et al. (2004), Obermeier et al. (2005) and Lee et al. (2006)
			CRC	Koo et al. (2015) and Luo et al. (2020)
			GVHD	Heimesaat et al. (2010)
			Human study	
			CRC	Gao et al. (2018) and Messaritakis et al. (2022)
NLR	–	–	–	
NOD1	IEC; mesothelial cell; MC; TC; BC	DAP-peptidoglycan of Gram-negative bacteria Certain Gram-positive bacteria (Hasegawa et al., 2006)	Mouse model	
			Neurological disorders	Layunta et al. (2018) and Pusceddu et al. (2019)
			CRC	Jiang et al. (2020), Maisonneuve et al. (2021) and Wei et al. (2022)
NOD2	Enterocyte, CSC, PC, M, GC (Naama et al., 2023); MC (Magalhaes et al., 2008)	Phosphorylated-MDP of bacteria ssRNA of virus	Mouse model	
			Ileitis/Colitis	Watanabe et al. (2006), Chu et al. (2016), Yadav et al. (2017) and Lin et al. (2020)
			CRC	Couturier-Maillard et al. (2013)
			Human study	
			IBD	Hampe et al. (2001), Ogura et al. (2001), Cleynen et al. (2013), Liu et al. (2015a) and Yadav et al. (2017)
NLRP3	IEC; MC (Ruiz et al., 2017)	LPS of bacteria; Pore-forming toxins; Nucleic acids of bacteria and virus; Unfermented dietary β-fructan fibers	Mouse model	
			Colitis	Bauer et al. (2010), Zaki et al. (2010) and Mehto et al. (2019)
			CAPS model	Lee et al. (2012) and Yao et al. (2017)
			Metabolic syndrome	Vandanmagsar et al. (2011) and Henao-Mejia et al. (2012)
			CRC	Allen et al. (2010), Guo et al. (2014), Qiao et al. (2020) and Shao et al. (2021)
			Human study	
			IBD	Zhou et al. (2021)
			CAPS	Hoffman et al. (2001), Aksentijevich et al. (2002) and Feldmann et al. (2002)
NLRP6	Enterocyte (Xing et al., 2021), GC; MC (Anand et al., 2012)	RNA of virus; LTA and LPS of bacteria; Taurine, histamine, spermine, SCFAs	Mouse model	
			Colitis	Elinav et al. (2011), Mukherjee et al. (2020) and Gao et al. (2023)
			Neuroinflammation	Li et al. (2019a), Matheis et al. (2020) and Muller et al. (2020)
			GVHD	Toubai et al. (2019)
			Metabolic syndrome	Henao-Mejia et al. (2012)
			Enterovirus infection	Wang et al. (2015a)
NLRP9b	Enterocyte	dsRNA of rotavirus	Mouse model	
			Rotavirus infection	Zhu et al. (2017b)
NLRP12	MC; TC (Lukens et al., 2015)	Ligands unknown *Lachnospiraceae*; *Yersinia pestis* (Vladimer et al., 2012); *Brucella abortus* (Silveira et al., 2017)	Mouse model	
			Colitis	Zaki et al. (2011), Allen et al. (2012), Lukens et al. (2015) and Chen et al. (2017)
			CRC	Zaki et al. (2011) and Allen et al. (2012)
			Metabolic syndrome	Truax et al. (2018)
NLRC3	IEC; Mф; TC	dsDNA of HSV-1 (Li et al., 2019c)	Mouse model:	
			Colitis	Karki et al. (2016)
			CRC	Karki et al. (2016, 2017)
NLRC4	IEC; MC	Flagellin, T3SS apparatus (rod protein) of bacteria	Mouse model	
			Colitis	Carvalho et al. (2012b)
			Rotavirus infection	Zhang et al. (2014a)
			CRC	Allam et al. (2015)
			Human study	
			IBD	Steiner et al. (2022)
			Metabolic syndrome	Furman et al. (2017)
RLR	Wide expression	–	–	
RIG-1		5ʹ-Triphosphorylated RNA, Short-chain dsRNA of virus	Mouse model	
			Colitis	Wang et al. (2007) and Liu et al. (2019)
			Rotavirus infection	Broquet et al. (2011) and Sen et al. (2011)
			CRC	Zhu et al. (2017a) and Song et al. (2022)
			GVHD	Fischer et al. (2017)
			Type 1 diabetes	Shigemoto et al. (2009)
MDA5		Long-chain dsRNA of virus	Mouse model	
			Colitis	Ratsimandresy et al. (2017)
			Rotavirus infection	Broquet et al. (2011) and Sen et al. (2011)
			Type 1 diabetes	Smyth et al. (2006) and Shigemoto et al. (2009)
			Human study	
			IBD	Huang et al. (2017) and Adiliaghdam et al. (2022)
–	–	–	–	
AIM2	IEC; MC; TC (Chou et al., 2021); BC (Yang et al., 2021); RBC (Kalantari et al., 2014)	dsDNA of bacteria, virus and parasites	Mouse model	
			Colitis	Ratsimandresy et al. (2017)
			Diabetes	Dang et al. (2017) and Leite et al. (2020)
			CRC	Man et al. (2015) and Wilson et al. (2015a)
			Human study	
			IBD	Vanhove et al. (2015)
			CRC	Mori et al. (2001)
			SBC	Schulmann et al. (2005)
CLR	–	–	–	
Dectin-1	IEC; Mф, DC, neu	β-Glucan of fungus	Mouse model	
			Colitis	Heinsbroek et al. (2012), Iliev et al. (2012), Tang et al. (2015b) and Wang et al. (2022a)
			Human study	
			UC	Iliev et al. (2012) and Sharifinejad et al. (2021)
Dectin-2	Mф (Yan et al., 2017), DC (Watanabe et al., 2023), neu	α-Mannan of fungus; Lipoglycans of bacteria	Mouse model	
			Colitis	Duan et al. (2021) and Wang et al. (2022a)
SIGNR3	MC	Fugus; SLPA from *Lactobacillus*	Mouse model	
			Colitis	Eriksson et al. (2013) and Lightfoot et al. (2015)
CGAS/STING	Wide expression	DNA of bacteria; DNA of virus	Mouse model	
			Intestine inflammation	Leibowitz et al. (2021), Shmuel-Galia et al. (2021) and Wang et al. (2022b)
			CRC	Hu et al. (2021) and Kunac et al. (2022)
			Obesity	Gao et al. (2022a)

IEC, intestine epithelial cell; PC, Paneth cell; GC, Goblet cell; CSC, crypt stem cell; M, M cell; MC, myeloid cell; Mф, macrophage; neu, neutrophil; ENS, enteric nervous system; DC, dentritic cell; TC, T cell; BC, B cell; RBC, red blood cell; CAPS: cryopyrin-associated periodic fever syndromes; SBC, small bowel cancer; GVHD, graft-versus-host disease; UC, ulcerative colitis; CRC, colorectal cancer; IBD, inflammatory bowel disease.

**Table 2.** Sensors of microbial metabolites in the intestine.

	Expression	Microbial signals	Diseases associated	
GPCR	–	–	–	–
GPR41	IEC; MC; ENS	SCFAs: propionate, butyrate, isobutyrate	Mouse model	
			Intestine inflammation	Kim et al. (2013) and Yang et al. (2020)
			Allergic inflammation	Trompette et al. (2014) and Zaiss et al. (2015)
			Metabolic syndrome	De Vadder et al. (2014), Tang et al. (2015a) and Kimura et al. (2020)
GPR43	IEC; ILC3; MC; mast cell; TC; BC	SCFAs: propionate, acetate	Mouse model	
			Intestine inflammation	Maslowski et al. (2009), Kim et al. (2013) and Sun et al. (2018b)
			Allergic inflammation	Maslowski et al. (2009), Macia et al. (2015), Tang et al. (2015a), Thorburn et al. (2015) and Tan et al. (2016)
			CRC	Chen et al. (2020a)
			Cardiovascular disease	Kaye et al. (2020)
			GVHD	Fujiwara et al. (2018)
			Metabolic syndrome	Mariño et al. (2017) and Kimura et al. (2020)
			Human study	
			CRC	Tang et al. (2011)
GPR109a	IEC; MC	SCFAs: niacin/vitamin B3, butyrate	Mouse model	
			Colitis	Singh et al. (2014) and Macia et al. (2015)
			CRC	Singh et al. (2014) and Chen et al. (2020a)
			GVHD	Docampo et al. (2022)
			Allergic inflammation	Tan et al. (2016)
			Cardiovascular disease	Lukasova et al. (2011) and Kaye et al. (2020)
GPR120	IEC; MC; TC	LCFAs: ω-3 fatty acids (Oh et al., 2010); ω-6 fatty acids (Mobraten et al., 2013)	Mouse model	
			Colitis	Yang et al. (2022)
			CRC	Rubbino et al. (2022)
			Metabolic syndrome	Hirasawa et al. (2005), Tanaka et al. (2008), Ichimura et al. (2012), Yan et al. (2013), Yore et al. (2014) and Paschoal et al. (2020)
TGR5	IEC; ENS (Poole et al., 2010); MC	Secondary bile acids; Oleanolic acid	Animal model	
			Colitis	Sinha et al. (2020), Sorrentino et al. (2020) and He et al. (2022)
			Digestive disease	Alemi et al. (2013)
			Metabolic syndrome	Thomas et al. (2009), Ding et al. (2016), Pathak et al. (2018), Chaudhari et al. (2021), Zheng et al. (2021) and Münzker et al. (2022)
AHR	IEC; MC; ENS; ILCs; TC	Trp metabolites; Bacterial pigments	Mouse model	
			Colitis	Goettel et al. (2016), Lamas et al. (2016), Iyer et al. (2018), Sanmarco et al. (2022) and Panda et al. (2023)
			Bacterial infection	Kiss et al. (2011), Li et al. (2011b, 2018), Qiu et al. (2012, 2013), Schiering et al. (2017) and Panda et al. (2023)
			Metabolic syndrome	Wada et al. (2016) and Natividad et al. (2018)
			CRC	Díaz-Díaz et al. (2016), Metidji et al. (2018) and Gronke et al. (2019)
			Neurological disorder	Rothhammer et al. (2018) and Obata et al. (2020)
Nuclear receptor	–	–		–
FXR	IEC; MC	Bile acids: CDCA, LCA, 3-keto-LCA, DCA, and CA	Mouse model	
			Intestine inflammation	Gadaleta et al. (2011), Zhou et al. (2014), Chen et al. (2022), Huo et al. (2022) and Pi et al. (2023)
			Coronavirus infection	Brevini et al. (2023b)
			CRC	Modica et al. (2008), Maran et al. (2009), Fu et al. (2019) and Yu et al. (2020)
			Metabolic syndrome	Sinal et al. (2000), Ryan et al. (2014), Fang et al. (2015), Jiang et al. (2015), Trabelsi et al. (2015), Pathak et al. (2018), Albaugh et al. (2019) and Münzker et al. (2022)
			Human study	
			CRC	Modica et al. (2010) and Lax et al. (2012)
			Metabolic syndrome	Sun et al. (2018a)
VDR	IEC; MC; TC; BC	Bile acids: LCA, 3-keto-LCA; Vitamin D [1,25(OH)2D3]	Mouse model	
			Intestine inflammation	Ooi et al. (2013), Du et al. (2015), Golan et al. (2015), Wu et al. (2015), He et al. (2018), Lu et al. (2020, 2021), Chatterjee et al. (2021) and Gao et al. (2023)
			CRC	Zhang et al. (2022)
			Human study	
			UC	Wada et al. (2009), Meckel et al. (2016) and Gao et al. (2023)
			CRC	Wada et al. (2009), Fernández-Barral et al. (2020), Messaritakis et al. (2020, 2022), Afshan et al. (2021), Latacz et al. (2021) and Zhu et al. (2021)

IEC, intestine epithelial cell; MC, myeloid cell; ENS, enteric nervous system; ILC, innate lymphoid cell; TC, T cell; BC, B cell; RBC, red blood cell; GVHD, graft-versus-host disease; UC, ulcerative colitis; CRC, colorectal cancer; LCA, lithocholic acid; DCA, deoxycholic acid; CDCA, chenodeoxycholic acid; CA, cholic acid.

### Inflammatory bowel disease

IBD represents a group of chronic inflammatory disorders that affect the colon, small intestine, and the extra-intestinal organs. UC and CD are the two most prevalent forms of IBD. There is increasing evidence that the gut microbiota plays a central role in the pathogenesis of IBD (Baumgart and Carding, 2007; Lloyd-Price et al., 2019). GWAS identified several IBD-related susceptibility genes that showed involvement of the gut microbiota in the pathogenesis of IBD. A meta-analysis of GWAS for the two most common forms of IBD, namely, CD and UC, identified 71 new associations and 163 IBD-related loci (Jostins et al., 2012). Furthermore, other large genome sequencing studies reported several IBD susceptibility loci in genes encoding PRRs and other factors across various human populations (Liu et al., 2015a; Somineni et al., 2021). *NOD2* gene locus is highly associated with IBD risk (Hampe et al., 2001; Hugot et al., 2001; Ogura et al., 2001). NOD2 is an intracellular receptor for MDP, which is found in all the bacteria. CD patients with NOD2 mutations showed significantly lower levels of antimicrobial peptides such as α-defensin (Wehkamp et al., 2004). *ATG16L1*, an abbreviation of autophagy related 16 like 1, is another well-established susceptibility gene for UC. The NOD1/NOD2/ATG16L pathway was critical for the pathogenesis of CD because both NOD2 and NOD1 recruited ATG16L to the site of bacterial entry and triggered autophagy (Cooney et al., 2010). Interaction between a specific enteric viral infection and a specific mutation in the *ATG16L1* locus determines susceptibility to IBD (Cadwell et al., 2010). Higher proportions of abnormal Paneth cells were associated with shorter time to recurrence of CD after surgery; moreover, the proportion of abnormal cells was associated with the cumulative numbers of *NOD2* and *ATG16L1* risk alleles (VanDussen et al., 2014). dl-Endopeptidase, a *Firmicutes* peptidoglycan remodeling enzyme, increased NOD2 levels in the gut and impacted colitis outcomes (Gao et al., 2022b). This study showed that depletion of dl-endopeptidase contributed to CD pathogenesis through NOD2 signaling. Supplementation of dl-endopeptidases or dl-endopeptidase-producing bacteria strains and a synthetic lipophilic analog of MDP protected against colitis in mice by restoring the NOD2 pathway. This provided a therapeutically modifiable target for the CD patients.

Changes in the fungal microbiota, an integral part of the complex microbial community, are also linked to IBD. Deep sequencing of the rRNA genes showed that the abundance of specific *Candida taxa* was increased in the IBD samples (Chehoud et al., 2015). The abundance and preferential localization of *Debaryomyces hansenii* in the incompletely healed intestinal wounds of mice and the inflamed mucosal tissues of human subjects with CD impaired mucosal healing through the myeloid cell-specific type 1 IFN–CCL5 axis (Jain et al., 2021). *Candida albicans*-secreted peptide toxin candidalysin significantly influenced inflammation and altered gut immunity in patients with IBD; moreover, *C. albicans* strains with a high immune cell damaging capacity induced intestinal inflammation through IL-1β-dependent mechanisms in the UC models (Li et al., 2022b). Intestinal fungi also promoted intestinal host-microbiota homeostasis. Mucosal fungi promoted host-protective immunity and epithelial barrier function through type 17 immunity (Leonardi et al., 2022). Therefore, host–fungal interactions are novel diagnostic and therapeutic targets for IBD.

### Type I diabetes

Autoimmune diseases are complex diseases caused by a combination of genetic, environmental, and life-style factors. Recent studies have demonstrated that the dysregulation of the composition and function of the gut microbiota are involved in autoimmune disorders such as type I diabetes (T1D) (Miyauchi et al., 2023). MyD88 deficiency leads to changes in the composition of the gut microbiota. Germ-free non-obese diabetic (NOD) mice with MyD88 deficiency developed severe diabetes, but colonization of these mice with defined gut microbiota attenuated diabetes (Wen et al., 2008). Non-synonymous mutants of human RIG-I (S183I) and MDA5 (E627*, I923V, and A946T) are implicated in the resistance against type I diabetes (Smyth et al., 2006; Shigemoto et al., 2009). AIM2^−/−^ mice treated with broad-spectrum antibiotics are protected from streptozotocin-induced T1D and lower pancreatic pro-inflammatory response, thereby implicating the role of gut microbiota in T1D (Leite et al., 2020). In obese subjects, intestinal microbial DNA-containing vesicles pass through the gut barrier and are transferred into islet β cells, resulting in enhanced inflammation and impaired insulin secretion through activation of the cGAS/STING signaling pathway (Gao et al., 2022a). Moreover, diabetic incidence is higher in the NOD mice under germ-free conditions than those under specific pathogen-free conditions. This finding was inconsistent with the higher prevalence of T1D is in countries with better hygiene (Bach, 2002; Vatanen et al., 2018; Kim et al., 2020).

### Metabolic syndrome

There is extensive evidence that the gut microbiota is important for the host in harvesting energy from the diet and increasing fat storage (Turnbaugh et al., 2006; Samuel et al., 2008). Germ-free mice ingested 29% more calories but showed 40% lower body fat than the conventional mice (Turnbaugh et al., 2006). The weight gain of the germ-free mice was lower and were protected against development of insulin resistance (Piya et al., 2013). In mice, administration of a specific protein isolated from the outer membrane of *A. muciniphila* interacted with TLR2 and prevented the development of obesity and associated complications (Plovier et al., 2017). A proof-of-concept study (Clinical trial No. NCT02637115) showed that supplementation with *A. muciniphila* improved several metabolic parameters associated with the obesity-related disorders but the overall structure of the gut microbiome was unaffected (Depommier et al., 2019). The gut microbiota contributed to metabolic disease through chronic low-grade inflammation (Gregor and Hotamisligil, 2011). For example, chronically increased plasma LPS concentration triggered inflammation-related obesity and metabolic disease in mice (Cani et al., 2007). The binding of LPS to TLR4 triggered NF-κB- and mitogen activated protein kinase (MAPK)-dependent secretion of pro-inflammatory cytokines (Neal et al., 2006). TLR4-deficient mice were protected against HFD-induced insulin resistance (Shi et al., 2006b). Furthermore, *ob*/*ob* mice with CD14 deficiency were unable to induce LPS signaling and were resistant to weight gain and insulin hypersensitivity (Cani et al., 2008). The gut microbiota also affects host immunity via other microbial-derived metabolites in addition to LPS. Indole is a microbial-derived metabolite that is produced by the bacterial tryptophanase, which is present in many commensal bacteria (DeMoss and Moser, 1969). Indole is metabolized into 3-indoxyl sulfate in the liver after absorption into blood from the intestine. Indoxyl sulfate is related to obesity because it promoted synthesis and secretion of the pro-inflammatory cytokine IL-6 through AhR (Ramadoss et al., 2005; Russell et al., 2013). The dysfunction of fatty acid and bile acid sensors also caused metabolic syndrome. The activation of GPR120, a lipid sensor, mediated the secretion of glucagon-like peptide-1 (GLP-1). GPR120 deficiency or mutations (p.R270H in the European populations) decreased insulin sensitivity and caused glucose intolerance and fatty liver, thereby increasing the risk of obesity in the mice models and humans (Hirasawa et al., 2005; Ichimura et al., 2012; Paschoal et al., 2020). TGR5 signaling pathway was triggered by the bile acids and played a significant role in regulating intestinal GLP-1 secretion (Thomas et al., 2009). Cholic acid-7-sulfate is an endogenous bile acid and a TGR5 agonist that enhanced glucose tolerance in the insulin-resistant mice by increasing GLP-1 secretion (Chaudhari et al., 2021). However, activation of another bile acid receptor, FXR in the enteroendocrine L cells decreased GLP-1 secretion by inhibiting glycolysis. Inhibition of FXR signaling in the intestine improved metabolic parameters in the mouse models of obesity (Jiang et al., 2015; Trabelsi et al., 2015). In the T2D mouse model, metformin acted partially through the *B. fragilis*—glycoursodeoxycholic acid (GUDCA)-intestinal FXR axis to improve metabolic dysfunction (Sun et al., 2018a). Conversely, a gut-restricted FXR agonist fexaramine robustly induced enteric fibroblast growth factor 15 (FGF15) and induced alterations in the bile acid composition and metabolic improvements without activating the FXR-target genes in the liver (Fang et al., 2015). This suggested that tissue restricted FXR activation was potentially a novel approach for treating obesity and metabolic syndrome.

### Neurological disorders

The gut microbiota also plays a significant role in the bidirectional communication along the “gut–brain axis”, which regulates the central nervous system development, function, and pathogenesis (Socała et al., 2021). Emerging evidence suggests that the NLRs and the TLRs in the gut–brain axis play a critical role in the pathogenetic mechanisms underlying neurodevelopmental and neurodegenerative disorders (Keogh et al., 2021). IEC-specific Nod1 knockout mice showed stress-induced anxiety-like behavior and cognitive impairment by affecting 5-HT biosynthesis and signaling (Pusceddu et al., 2019). Microbiota-derived LPS promoted survival of enteric neurons and gastrointestinal motility in mice by activating TLR4 and NF-κB (Anitha et al., 2012). SNPs including TLR1 rs4833095, TLR2 rs3804099 and TLR4 rs1927914 were associated with increased risk of Parkinson’s disease (PD) (Zhao et al., 2015; Gorecki et al., 2020). Precursors of neurotransmitters like serotonin, GABA, dopamine, and norepinephrine are generated by the gut microbiota. Serotonin, dopamine, and norepinephrine act on the ENS through specific receptors in the brain, whereas transporter for GABA is expressed in the blood–brain barrier (Lyte, 2011; Reardon, 2014; Dinan et al., 2015; Hubbard et al., 2015). Amyloid protein in the gut bacteria demonstrate molecular mimicry and can elicit cross-seeded misfolding, inflammation and cellular cytotoxicity that initiates or influences the pathogenesis underlying PD, Alzheimer’s disease (AD), and other neurological disorders (Friedland, 2015). Bacterial amyloid proteins induce molecular mimicry pathways via TLR1/2 signaling, which enhanced inflammatory responses to brain amyloid proteins such as α-synuclein (Oppong et al., 2013; Chen et al., 2016; Sampson et al., 2020). The upregulation of TLR2 signaling in the microglia was associated with neuroinflammation in PD (Beraud and Maguire-Zeiss, 2012). Metabolites of dietary Trp produced by the commensal flora activated AHR and suppressed CNS inflammation due to pathological activities of the microglia and the astrocytes in the experimental autoimmune encephalomyelitis (EAE) mouse model of multiple sclerosis (Rothhammer et al., 2018). In the intestinal neural circuits, AHR is a microbiota-related biosensor that impacts intestinal motility (Obata et al., 2020).

### Cancer

Colorectal cancer (CRC) is a disease of the ISCs that is characterized by dysregulation of multiple signaling pathways and components in the intestine. Inflammation associated with IBD, heritable genetic defects, diets with low fiber content and high red meat content, and imbalance in the gut microbiota are known risk factors of CRC (Janney et al., 2020). The expression of TLR2 is significantly higher in the tumor tissues compared with the adjacent normal intestinal tissues of the CRC patients. TLR2 knockdown significantly inhibited proliferation of the CRC cells but exacerbated colitis symptoms in the mouse colitis-associated cancer model (CAC) (Meng et al., 2020). Epigenetic modifying drugs are used to treat several cancers. A recent study showed potential immunoregulatory role of these drugs in response to viral stimulation, especially changes in the TLR3 responses (Hennessy et al., 2019). *Peptostreptococcus anaerobius* activated TLR2 and TLR4 on the colon cells and increased the intracellular levels of reactive oxygen species (ROS), which promoted cholesterol synthesis and cancer cell proliferation. *Peptostreptococcus anaerobius* levels were higher in the human CRC tissues and adenomas compared to the non-tumor tissues (Tsoi et al., 2017). Pro-inflammatory effects induced by the activation of TLRs promoted malignant progression of colon cancer (Yesudhas et al., 2014; Gao et al., 2018; Moradi-Marjaneh et al., 2018). Furthermore, infections promoted cancer progression by enhancing tumor invasiveness through the TLR9-mediated matrix metalloproteinases (MMPs) (Merrell et al., 2006). However, IRAK-M, a negative regulator of TLR signaling, was expressed in the CRC cells through combined TLR and Wnt activation and promoted CRC cell proliferation by stabilizing the STAT3 protein (Kesselring et al., 2016). These conflicting data may be due to the negative feedback of the pro-inflammatory signaling pathways related to the TLRs. Furthermore, TLR5-dependent commensal microbiota drives the malignant progression of tumors by increasing IL-6 levels and depletion of the commensal bacteria abrogates TLR5-dependent tumor progression. However, TLR5 agonists acted as organ-specific immunoadjuvants and enabled efficient anti-tumor vaccination by stimulating the NK-DCs-CD8^+^ T cell axis (Brackett et al., 2016). NOD1 is another PRR that is highly expressed in the human CRC tissues and the human and murine CRC cell lines and augments CRC cell metastasis through the p38 MAPK pathway (Jiang et al., 2020). *AIM2* gene mutations were frequent in patients with intestinal cancers (Schulmann et al., 2005). AIM2 suppressed uncontrolled expansion of ISCs by dysregulating the Wnt signaling pathway. In the CAC model, AIM2-deficient mice developed significantly higher number of colon tumors that were mediated by DNA-PK-Akt activation but were independent of inflammasome activation (Man et al., 2015; Wilson et al., 2015a). Epithelial metabolite receptors, such as GPR120, VDR and AHR, are essential for maintaining the mucosal barrier integrity, restoring the gut barrier homeostasis, and preventing CRC development (Metidji et al., 2018; Rubbino et al., 2022; Zhang et al., 2022). Several VDR gene polymorphisms are significantly associated with colorectal cancer development and progression (Latacz et al., 2021; Messaritakis et al., 2022).

## Outlook

In the recent decades, several studies have investigated the gut microbial sensing and shown that co-evolution of the gut microbiota and the immune system plays a pivotal role in human health and disease. However, the relationship between the gut microbiota, microbiota-derived metabolites, and the host immune system is far from being understood fully because of the extensive size of the gut microbiota and the limitations of current technology. The mechanistic details of many microbial sensors have been discovered. However, studies have also reported controversial results about the roles of the microbial sensors in the intestine. For example, TLR4 signaling reinforced by LPS from the *Eubacterium rectale*-deficit microbiota promoted intestinal inflammation. However, TLR4 ameliorated colitis by upregulating Treg cells by interacting with *A. muciniphila* (Liu et al., 2022b; Lu et al., 2022). These bidirectional roles of TLR4 in the intestinal inflammation may be due to the structural differences in the microbial components such as LPS. In 2022, a study reported that NOD2 recognized phosphorylated MDP instead of unmodified MDP (Stafford et al., 2022). Therefore, precise elucidation of the structure and functions of the microbial ligands is necessary to determine the exact microbe-host signaling connections. Recent studies have investigated the mechanisms by which the commensal microorganisms modulate the innate immune sensors using high throughput bacterial screening and the mechanisms by which complex dietary or microbe-derived metabolites modulate the host receptors (Cohen et al., 2017; Spindler et al., 2022). Furthermore, in-depth studies are necessary to determine the exact role of several orphan ligands or receptors including NLR family members such as NLRP12 and other orphan GPCRs. Thus, the “one microbe, one response” approach needs to be replaced by systematic studies. Moreover, development of advanced computational tools and other integrative technologies are needed for unraveling the mechanisms underlying the host-microbiota relationship, especially in relation to human health and disease.

## Abbreviations

Aβ: amyloid-β
AD: Alzheimer’s disease
AHR: aryl hydrocarbon receptor
AID: activation-induced cytidine deaminase
AIM2: absence in melanoma 2
ALR: AIM2-like receptor
AOM: azoxymethane
AP: adaptor protein
APC: antigen presenting cell
ATG16L1: autophagy related 16 like 1
BMP: bone morphogenetic protein
CAC: colitis-associated cancer model
CAPS: cryopyrin-associated periodic syndrome
CARD: caspase recruitment domain
CART: cocaine- and amphetamine-regulated transcript
CD: Crohn’s disease
cGAMP: cyclic-di-GMP/AMP
cGAS: cyclic GMP-AMP synthase
CLR: C-type lectin receptor
CRC: colorectal cancer
CYLD: CYLD lysine 63 deubiquitinase
DAMPs: danger-associated molecular patterns
DAP: d-glutamyl-meso-diaminopimelic acid
DC: dendritic cell
DHX15: DEAH-box helicase 15
DRG: dorsal root ganglion
DSS: dextran sulfate sodium
EAE: experimental autoimmune encephalomyelitis
ECMV: encephalomyocarditis virus
EEC: enteroendocrine cell
ENS: enteric nervous system
ER: endoplasmic reticulum
FGF15: fibroblast growth factor 15
FOS: oligofructose
FXR: Farnesoid X receptor
GALT: gut-associated lymphoid tissue
GP-BAR1: G protein-coupled bile acid receptor, also named as TGR5
GPCR: G protein-coupled receptor
GUDCA: glycoursodeoxycholic acid
GVHD: graft-versus-host disease
GWAS: genome-wide association study
5-HT: 5-Hydroxytryptamine
HFD: high-fat diet
HLCs: hepatocyte like cells
HMGB1: high mobility group box 1 protein
HMP: Human Microbiome Project
IBD: inflammatory bowel disease
IEC: intestinal epithelial cell
IEL: intraepithelial lymphocyte
IFN: interferon
IgA: Immunoglobulin A
IGF-1: insulin-like growth factor-1
IL: interleukin
ILCs: innate lymphoid cells
ILF: isolated lymphoid follicle
ISG: IFN-stimulated gene
LCFA: long-chain fatty acid
LGP2: laboratory of genetics and physiology 2
LLPS: liquid–liquid phase separation
LPS: lipopolysaccharide
LRR: leucine-rich repeat
LTA: lipoteichoic acid
LTi: lymphoid tissue-inducer
M cells: microfold cells
MAPK: mitogen activated protein kinase
MAVS: mitochondrial antiviral signaling protein
MCFAs: medium-chain fatty acids
MDA5: melanoma differentiation-associated gene 5
MDP: muramyl dipeptide
MHC: major histocompatibility complex
MMP: matrix metalloproteinase
MNV: murine norovirus
MRGPRX2: MAS related GPR family member X2
MRI: magnetic resonance imaging
mTOR: mechanistic target of rapamycin kinase
MyD88: myeloid differentiation factor 88
NAGK: *N*-acetylglucosamine kinase
NAIP: NLR family apoptosis inhibitory protein
NACHT: NAIP, CIITA, HET-E and TP1
N-C11-G: *N*-undecanoylglycine
NF-κB: nuclear factor kappa-B
NK: natural killer
NLR: NOD-like receptor
NLRC4: NLR family, CARD domain-containing 4
NLRP3: NLR family, pyrin domain-containing 3
NOD model: non-obese diabetic model
NOD1: nucleotide-binding oligomerization domain-containing 1
NRF2: nuclear factor erythroid 2-related factor 2
PAMPs: pathogen-associated molecular patterns
PD: Parkinson’s disease
PI3K: phosphatidylinositol-3-kinase
PRRs: pattern recognition receptors
PSC: pluripotent stem cell
PXR: Pregnane X receptor
PYD: pyrin domain
RAF-1: v-raf-1 murine leukemia viral oncogene homolog 1
RAR: retinoic acid receptor
RIG-I: retinoic acid-inducible gene I
RIP2: receptor-interacting-serine/threonine-protein kinase 2
RLR: RIG-I-like receptor
RORγt: retinoic acid receptor-related orphan receptor-γt
ROS: reactive oxygen species
RV: rotavirus
RXR: retinoid X receptor
SCFAs: short-chain fatty acids
sIgA: secretory IgA
SIGNR3: specific intracellular adhesion molecule-3 grabbing non-integrin homolog-related 3
SNP: single nucleotide polymorphism
SPF: specific pathogen free
STING: sequestering and inhibiting the stimulator of IFN genes
T1D: type I diabetes
T3SS: Type III secretion systems
TIR: Toll/IL-1 receptor
TJ: tight junction
TLR: Toll-like receptor
Treg: regulatory T cell
TRIF: TIR domain-containing adaptor inducing IFNβ
Trp: tryptophan
Trpa1: transient receptor potential ankyrin A1
UC: ulcerative colitis
UDCA: ursodeoxycholic acid
VDR: Vitamin D receptor
WAT: white adipose tissue
XIAP: X-linked inhibitor of apoptosis protein
